# Recent advances in bio-microsystem integration and Lab-on-PCB technology

**DOI:** 10.1038/s41378-025-00940-4

**Published:** 2025-05-08

**Authors:** Sotirios Papamatthaiou, Pavlos Menelaou, Bilal El Achab Oussallam, Despina Moschou

**Affiliations:** Department of Electronic and Electrical Engineering, University of Bath, Bath, BA2 7AY UK

**Keywords:** Chemistry, Electrical and electronic engineering

## Abstract

The concept of micro-total analysis systems (µTAS) introduced in the early 1990s revolutionized the development of lab-on-a-chip (LoC) technologies by miniaturizing and automating complex laboratory processes. Despite their potential in diagnostics, drug development, and environmental monitoring, the widespread adoption of LoC systems has been hindered by challenges in scalability, integration, and cost-effective mass production. Traditional substrates like silicon, glass, and polymers struggle to meet the multifunctional requirements of practical applications. Lab-on-Printed Circuit Board (Lab-on-PCB) technology has emerged as a transformative solution, leveraging the cost-efficiency, scalability, and precision of PCB fabrication techniques. This platform facilitates the seamless integration of microfluidics, sensors, and actuators within a single device, enabling complex, multifunctional systems suitable for real-world deployment. Recent advancements have demonstrated Lab-on-PCB’s versatility across biomedical applications, such as point-of-care diagnostics, electrochemical biosensing, and molecular detection, as well as drug development and environmental monitoring. This review examines the evolution of Lab-on-PCB technology over the past eight years, focusing on its applications and impact within the research community. By analyzing recent progress in PCB-based microfluidics and biosensing, this work highlights how Lab-on-PCB systems address key technical barriers, paving the way for scalable and practical lab-on-chip solutions. The growing academic and industrial interest in Lab-on-PCB is underscored by a notable increase in publications and patents, signaling its potential for commercialization and broader adoption.

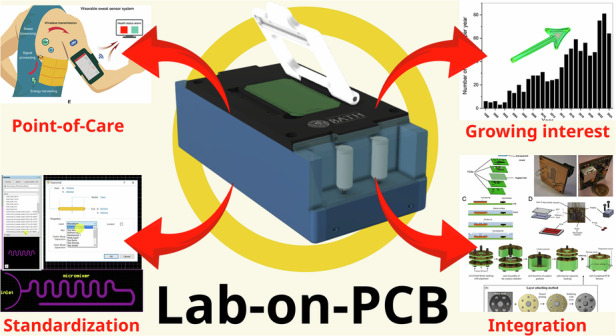

## Introduction

Since the introduction of the micro-total analysis system (µTAS) concept in the early 1990s^1^, the field of lab-on-a-chip (LoC) technologies has gained significant attention due to its potential to revolutionize conventional laboratory processes. LoC platforms, which integrate microfluidics and biosensing, aim to miniaturize and automate complex laboratory functions into a single, portable device. These advancements offer numerous benefits, including reduced sample volumes, rapid response times, and cost-effective operation, positioning LoC systems as powerful tools in diagnostics, drug development, and beyond^2^.

Despite substantial research efforts and investments, however, the commercial adoption of LoC technologies has remained far below its expected potential^3^. This is largely due to challenges related to scaling these systems for real-world applications. Traditional LoC platforms, which typically utilize materials like silicon, glass, and polymers, face limitations in terms of integration, standardization, and electrical interfacing. While these substrates are effective for certain tasks, they often fall short in supporting the multifunctional integration required for true µTAS devices, which demand robust and scalable electrical and fluidic interfacing to macroscopic systems, cost-effective and standardized mass production, and seamless integration of multiple microanalytical components.

To address these limitations, Lab-on-Printed Circuit Board (Lab-on-PCB) technology has emerged as a promising alternative^4^. PCBs, long established in the electronics industry for their reliable, low-cost manufacturing and excellent scalability, are increasingly being recognized for their potential in the development of LoC devices. First proposed in the late 1990s^5^, Lab-on-PCB leverages the multi-layer architecture and precise fabrication techniques inherent to PCBs, enabling researchers to explore new possibilities for integrating microfluidics, sensors, and actuators within a seamlessly integrated platform. Moreover, the versatility of PCBs allows for the incorporation of electrical components alongside microchannels, allowing more complex, multifunctional Lab-on-PCB systems suitable for high-throughput mass production^4^.

In recent years, the academic and industrial interest in Lab-on-PCB has grown exponentially, as demonstrated by the increasing number of publications and patents in this area, reflecting its growing acceptance in the research community as a viable solution for overcoming the technical barriers faced by traditional LoC systems. Researchers have successfully integrated microfluidics, sensors, and actuators onto PCB substrates, enabling a wide range of biomedical applications, such as point-of-care diagnostics, electrochemical biosensing, and molecular detection. Additionally, the utilization of Lab-on-PCB devices has been expanded to various fields, including drug development, environmental monitoring, and MEMS-related devices. As a result, the number of PCB-based LoC publications has seen a notable increase, and the potential for commercialization of these technologies is attracting further interest.

Previous reviews have covered the technical perspective of Lab-on-PCB and its capabilities to revolutionize lab-on-chip^4,6–8^. This review aims to explore the applications and impact of Lab-on-PCB technology within the research community in the past 8 years, demonstrating how it has transformed research and development since our last review in 2017^4^ when the term Lab-on-PCB had just been first introduced. By examining the latest advances in PCB-based microfluidics, biosensing and device integration along with the broader adoption of PCB-based systems, we will highlight how this technology progresses both the scalability and practical deployment of lab-on-a-chip solutions in real-world scenarios.

## Integrated Lab-on-Chip state-of-the-art

### Performance progress meets commercial upscaling bottleneck

LoC boasts impressive performance in terms of sensitivity for a wide analyte and medium range employing every transduction available system: electrical, electrochemical, optical and mechanical^9^. As an illustration, a self-powered, piezoelectric meter was presented for real-time blood glucose monitor in vivo^10^, an optical, portable and real-time salivary urea testing system was developed requiring only 1 μl of saliva with good correlation to a commercial system^11^, an immunoassay for early diagnosis of respiratory disease based on detection of tumor necrosis factor α, drastically reduces the analysis time to about 30 min, as opposed to hours in conventional methods^12^ and impedance cytometry is emerging as an electrical, label-free and high-throughput method to segment cellular systems^13^.

Despite the achieved impression on the performance level, the LoC field has not yet fulfilled its potential in the research or clinical areas, not to mention the most crucial and fundamentally necessary commercialization level. A prominent example, particularly highlighted during the COVID-19 pandemic, was the reliance on lateral flow assays. Despite their widespread use, these assays lacked the necessary reliability and sensitivity to significantly curb the spread of the virus. Unfortunately, they represented one of the handful of tools in point-of-care diagnostics during that critical period. The disparity between the immense potential of LoC technology and the current reality of commercialized products can be attributed to the following critical factors:

- The integration aspect of lab-on-chip technology has not garnered sufficient attention from researchers, as many studies tend to concentrate primarily on the sensing components. Consequently, there has been limited emphasis on developing robust systems integrating liquid handling with electronics, which is critical for full device functionality and autonomy. The field would benefit greatly from a material that can seamlessly integrate the various stages of the laboratory process in a comprehensive and holistic manner. Such material should be capable of encompassing all necessary tasks without relying on conventional macroscale apparatus to handle essential functions externally. This would address a critical need to ensure lab-on-chip systems are truly self-contained in a sample-to-answer operation.
- One of the major limitations is the cost barrier to designing, prototyping, and manufacturing these devices at scale for widespread use. Most researchers concentrate on innovation and development, with limited emphasis on scaling up, practical applications, or cross-disciplinary collaborations. This area requires further investigation to bridge the gap between research advancements and real-world implementation;
- The lack of standardization discourages major investors and established industry players from supporting LoC technologies, as they perceive the likelihood of achieving significant profits only in the long term. The process typically begins in academic laboratories, and it can take an average of $34 million and six years to bring a point-of-care diagnostic device to market in the U.S^14^. However, venture capitalists often expect market potential and returns within a much shorter timeframe.

The limitations of various materials used in LoC fabrication were comprehensively discussed in our previous review (2017). Since then, no significant breakthroughs have emerged in achieving a true representation of a fully integrated LoC system. In summary, while silicon is preferred for its semiconducting properties and versatility in surface engineering, it faces challenges in optical detection due to its inherent opacity. Economically, silicon is not an ideal choice for fabricating LoC devices, as their larger physical dimensions compared to CMOS circuits lead to disproportionate material and production costs, limiting its use to merely single components. Photolithography, although capable of high-resolution fabrication, is similarly constrained by its expense, limiting its feasibility in resource-limited settings. Glass, with its transparency and biocompatibility, offers superior analytical capabilities but shares silicon’s drawbacks in terms of high production costs and the necessity of hazardous chemicals like hydrogen fluoride in its processing. Furthermore, both glass and silicon are rigid materials, making them unsuitable for applications such as cell culture, which requires gas permeability for sustained biological activity. Polymers like elastomers and thermoplastics provide a more economical alternative, offering advantages such as optical transparency and ease of fabrication. Polydimethylsiloxane (PDMS), a commonly used elastomer, is especially valued for its flexibility, gas permeability, and ease of bonding. It continues to be a widely used material in microfluidics research due to its versatility and ease of use, making it an ideal choice for developing new designs, rapid prototyping, and student training. It serves as an excellent platform for studying cell behavior and understanding the technological challenges associated with microfluidic systems, such as channel expansion, leakage, and clotting issues. However, techniques like soft lithography, despite being effective for rapid prototyping, are not easily scalable for mass production. Emerging technologies aimed at developing microfluidic platforms on flexible substrates are gaining traction, but integrating all essential components for fluid handling, signal transmission, and power supply into a single, fully functional system remains an ongoing challenge.

Emerging alternatives to traditional micro-fabrication methods for LoC devices, such as injection molding and 3D printing, offer promising advancements in scalability and cost-efficiency^14^. Injection molding-based fabrication of microfluidics presents a scalable solution for reducing the cost of device production, particularly when optimized for high-volume manufacturing. However, the initial costs associated with this method are substantial, primarily due to the complexities involved in master-mold design. This approach is most effective for features around 100 micrometers or larger, though advancements are being made to accommodate smaller feature sizes. Micro-injection molding has recently demonstrated promise in replicating microstructures, but further research on mold materials, tooling technologies, and both molding and demolding processes is necessary to improve repeatability and achieve resolutions in the micrometer range. 3D printing is advantageous due to its accessibility, lower cost for rapid prototyping, and widespread availability, allowing for the creation of custom designs. Additionally, the development of bio-compatible printing materials offers new possibilities for mimicking biological environments. Despite these benefits, 3D printing is currently unsuitable for cost-effective, high-volume production of single-use cartridges or consumables. Given the high costs associated with establishing large-scale microfluidic device fabrication infrastructure, the employment of more than one fabrication methods for different production stages may be needed; injection molding may be used when the design is finalized and is ready for mass production but initially 3D printing, hot embossing or elastomer casting may be useful for small quantities production for the clinical and feasibility validation due to the high cost associated with design modification with injection molding.

### Lab-on-PCB expansion

**Fig. 1:**
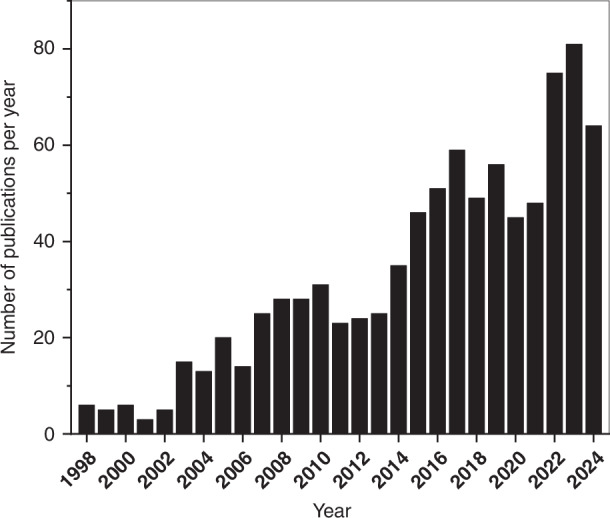
Number of published articles on PCB-based biosensors and LoC over 1998–2024. Scopus search date: 10/08/2024, Keywords used: “printed circuit board” + “microfluidics” or “printed circuit board” + “biosensor” or Lab-on-PCB

Since 2017, few researchers have explored the progress in the integrated LoC field^15,16^, with the vast majority of the review articles in the field focus on specific applications, e.g. virus detection^17^ or space applications^18^, or other niche aspects, e.g. carbon nanotubes in LoC^19^. In these last 8 years, nine review articles/book chapters^3^^,^^6–8^^,^^20–24^ covered the progress of Lab-on-PCB while the growth each year in the number of original research articles related to Lab-on-PCB has been striking, as depicted in Fig. 1. The shift in the number of review papers from integrated LoC to Lab-on-PCB since 2017 suggests a key trend in the research community: growing interest in the potential of this technology to enhance the integration of electronic and microfluidic systems, advancing LoC into a standardized, scalable integration platform.

LoC has been an active area of research for decades, with significant advancements in microfluidics, diagnostics, and point-of-care systems. By 2017, the fundamental principles and potential applications of LoC were well understood, and many key challenges, such as integration, miniaturization, and commercialization, were already addressed in earlier reviews. This could mean that fewer new ground-breaking developments were available to justify frequent reviews on integrated systems, leading to a natural decline in the publication of new review papers. LoC technology might have reached a more stable, mature stage where incremental improvements or specific niche applications dominate rather than broad innovations.

As the PCB industry has matured and become standardized, it offers a cost-effective, scalable, and reliable platform for combining electronics with fluidic components. The rise of review papers that discuss the capabilities and achievements of Lab-on-PCB indicates that the research community is shifting towards technologies that facilitate broader commercial applications and ease of production. Researchers are focusing on leveraging standardized PCB manufacturing processes to make LoC more accessible, particularly for point-of-care diagnostics and portable medical devices. The focus on Lab-on-PCB in review papers suggests an increasing emphasis on commercial feasibility and the development of systems that can be more easily mass-produced and integrated into existing infrastructures. Lab-on-PCB is seen as a solution for overcoming the bottlenecks in LoC commercialization, such as affordability, standardization, and ease of production. The merge of traditional microfluidic LoC systems with electronic circuits, inevitably expands interdisciplinary research that brings together fields like electronics, biotechnology, and materials science. The growth of review papers on Lab-on-PCB reflects increased interest from engineers and technologists working at the intersection of these fields, who see Lab-on-PCB as a platform for next-generation biosensors and diagnostic tools.

## Lab-on-PCB integration approaches

In this section, we aim to critically report and evaluate the fabrication and bonding processes presented for the integration of microfluidic structures and electronic components into integrated microanalytical Lab-on-PCB micro-systems. Components in our study can be considered any microfluidic, electronic or other component comprising a biochemical analytical function, such as reagent mixing, delivery, detecting and heating.

**Fig. 2:**
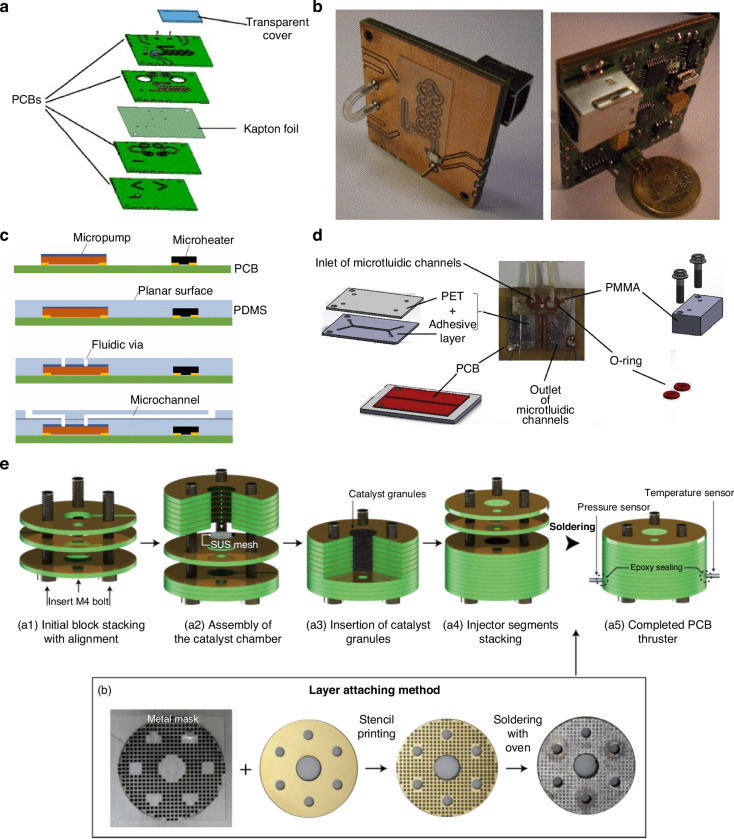
Overview of the Lab-on-PCB integration approaches. **a** Schematic of the seamless approach, showing the exploded view of the PCB stack of a flow injection analysis system (reproduced from ref. ^226^ copyright 2007, with permission from Elsevier). **b** Optical photograph of the PCB-Flow injection analysis seamless Lab-on-PCB example (reproduced from ref. ^226^ copyright 2007, with permission from Elsevier). **c** Diagram of the Stacked-up hybrid approach, for the fabrication of a surface mount electroosmotic pump and microfluidic integration PCB (reproduced from ref. ^65^, copyright 2008, with permission from IEEE). **d** Separate hybrid approach, schematic illustration of a microfluidic biofuel cell (reproduced from ref. ^95^, copyright 2020, with permission from Elsevier). **e** Structure of a Modular approach, showcasing a Lego-like fabrication process of PCB thruster (reproduced from ref. ^153^, copyright 2024, with permission from Elsevier)

We define three different types of Lab-on-PCB platforms, with the main criterion being the level of integration of different electronic and microfluidic components. The three types can be defined as seamless, hybrid, and modular. The seamless approach can be explained by the construction of microfluidic structures directly on the PCB substrate, while the hybrid approach is used to design and fabricate PCB/electronic and polymer/microfluidic layers separately. The hybrid type can be categorized as stack-up and separate based on the merging approach of the extrinsic, electronic, and microfluidic layers. Sequentially, building on the PCB substrate can be defined as stack-up, while individually building before bonding for the assembly of the device describes the separate type. Modular Lab-on-PCB consists of separately fabricated blocks, which can be combined in different orientations depending on the intended application. An overview of the different Lab-on-PCB approaches is depicted in Fig. 2.

### Seamless Lab-on-PCB

When PCB was suggested as a LoC substrate, the integration of different LoC components occurred by the construction of microchannels directly on the PCB substrate along with the sensors and electronics. This type can be defined as the seamless approach, stemming from the already developed PCB fabrication techniques adjusted to accommodate the inclusion of micromechanical structures on PCB materials integrating as such various microfluidic, electronic components on PCB substrates.

- **Copper traces, welding**

The concept involves the reconstruction of the cavities between copper lines into microchannels. The device and channels are then sealed usually with PDMS or other adhesives. The method is still being demonstrated, despite less often as the fabrication techniques have progressed, with Wang et al.^25^ defining the height and width of microchannels by the thickness of the copper layer and distance between adjacent copper structures, fabricated using lithography and etching. A PDMS lid was bonded after the surface was plasma treated. An on-chip electrolyte regulator was thus proposed on this platform to analyze diffusion properties in laminar flow.

Seamless integration of electronics and microfluidics via lithography was also demonstrated by the fabrication of interdigitated electrodes (IDEs) onsingle-sided copper clads via lithography and etching, utilizing the solder mask layer to form microwells around the working electrodes of the sensor used for Vimentin, a Potential Biomarker for Ovarian cancer^26^.

- **3D printed channels**

The use of 3D techniques has also been shown to seamlessly integrate microfluidics on PCB substrates. Using a direct ink writing 3D printer, fast-curing flexible silicone resin was deposited on commercial Arduino PCBs to form microchannels^27^ sealed with a top sheet clamped in place. The channel was evaluated by using the conductive fluid as a switch to turn on the LEDs on the PCB.

**Fig. 3:**
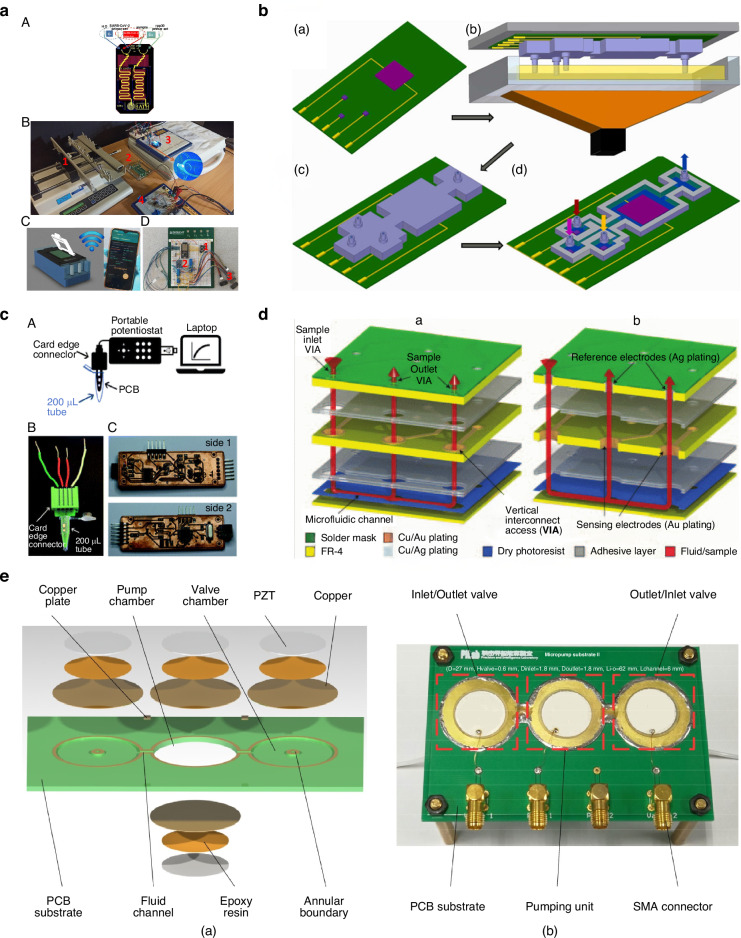
Seamless Lab-on-PCB approsache. **a** LoCKAmp device for SARS-CoV-2 detection with commercial microfluidic layer and embedded copper microheaters, reproduced from ref. ^55^, copyright 2023, with permission from Royal Society of Chemistry. **b** An integrated Lab-on-PCB via SLA 3D printing for integrating fluidic components, reproduced from ref. ^28^, copyright 2022, with permission from Elsevier. **c** Schematics and pictures of electroanalytical device of card edge connector, fitting inside a 200 µL tube, reproduced from ref. ^48^, copyright 2022, with permission from Royal Society of Chemistry. **d** Exploded view of the architecture of a Lab-on-PCB used for a microfluidic diluter and an electrochemical biosensor, reproduced from ref. ^51^, copyright 2016, with permission from IEEE. **e** Multi chamber piezoelectric pump based on pumping unit with double CPUAs, reproduced from ref. ^35^, copyright 2021, with permission from IOP Publishing

Stereolithography (SLA) printing has been used with photocurable resin-silica composites (Fig. 3b) of low coefficient thermal expansion^28^. This approach addressed the challenges of poor adhesion of SLA resins to PCB due to thermal expansion during temperature cycling. Compared with existing fabrication tools for microfluidic components (e.g. PDMS molding, micromachining, and other 3D printing techniques), the SLA route showed the possibility of one-step manufacturing for 3D hollow structures (number > 50) and the reliability of dissimilar material bonding (leakage test: all samples passed, number > 50; thermal cycling test: 90% samples made by formulation C survived 1000 cycles). The results showed that the method is promising and could extend the SLA potential in fabricating other Lab-on-PCB devices.

Gassman et al. present a rapid prototype technique, vat polymerization with an LCD as mask, also known as masked stereolithography (mSLA)^29^. These printers are available with resolutions down to 35 µm. Copper electrodes were fabricated on PCB substrates via standard etching techniques, before depositing the liquid resin via SLA printing. The liquid resin was then partially polymerized by light with the use of masks to protect the electrode areas. Layer by layer, the resin is polymerized, moving the masks further from the electrodes with each layer. In this study, the color mix resin basic from 3Djake was used which needs longer exposure times than other resins; however, this longer exposure time leads to less light bleed and less over-exposure for the base layers, which makes it suitable for printing small cavities. Afterwards, the uncured resin was washed off with isopropanol before the final curing to seal the channels, creating as such microfluidic cavities around the electrodes. An acrylic resin was also used to embed microfluidics on a PCB via SLA printing^30^. Following the step-by-step control of the curing layers, with the first curing layer being adjusted at a lower light intensity for a longer time to ensure adhesion between microfluidic and PCB layer. Masks were not used in this instance, and the layer covering the electrodes was not cured by exploiting the light intensity and time of curing. The following layers were cured at a faster pace and higher light intensity. The device was used as a microvalve, by the injection of liquid metal (LM) in the 3D printed, caterpillar-shaped microchannel. Electrochemical actuation of the Gallistan LM plug was achieved by filling the chambers with NaOH, which when under applied electric field moves from CLOSED to OPEN state, allowing fluid to flow perpendicular to the LM plug movement axis.

Lall et al.^31^ use additive technologies to print an integrated wearable biosensor patch with the circuits, encapsulations, body temperature sensor, humidity sensor, pulse-oximetric (pulse-Ox) sensor and electrodermal activity (EDA) sensor on an integrated, PCB-based wearable biosensor. Electrically conductive adhesive (ECA) pads were also printed to attach the power/control components.

- **Machining/milling/welding**
  Machining processes were also demonstrated for the integration of microfluidics and electronics. Micro milling channels on commercial polymeric substrates with embedded Cu microheaters, followed via polymeric adhesive sealing a miniaturized isothermal DNA amplification platform was produced^32^. Skotadis et al.^33^ fabricated a nanoparticle strain sensor by sealing the micro-milled channels using a flexible polyolefin film, with a silicone-based adhesive. Platinum nanoparticle networks, formed via a modified sputtering technique, self-assembled on the polyolefin layer to act as the flow sensor. The sensor detects flow by measuring changes in resistance, which occur due to strain in the polyolefin layer as fluid passes through the channel. This design achieved a competitive detection limit of 5 µL/min with a strain sensitivity of 0.021 (µL/min)^–1^.
  Another machining process that was utilized for Lab-on-PCB is welding. Wang et al. fabricated PCB process–based piezoelectric microfluidic pump that can linearly pump in and pump out of fluid with self-injection. The flow rate and back pressure can be controlled by changing the peak-to peak value, frequency, and phase difference of the driving voltages (Fig. 3e). The pumps were fabricated using PCB substrate with annular boundary and valve pads, welding the boundary components (inlet/outlet), the chamber walls and finally the circular piezoelectric unimorph actuators (CPUA)^34^. Utilizing the same structure and process, a few years later they demonstrated the bidirectional fluid with high control accuracy by arranging the two active piezoelectric valves on either side of the pumping unit^35^.
- **Sensor integration**
  The integration of Lab-on-PCB has also transferred to the realization of electrolyte-gated field-effect transistors (FET) devices, which conventionally require external reference electrodes, based on planarized, commercial PCB platforms. Papamatthaiou et al. demonstrate this for the first time by graphene printed, planar BioFET for DNA quantification^36^. It is the first PCB-based FET, utilizing the core board as the substrate and the PCB electrodes as the transistor drain, source and gate pads. Similarly, Fenech-Salerno et al.^37^ spray-coat IDE electrodes with graphene, with the reference electrode integrated on the planar PCB via Ag/AgCl paste for the electrode deposition.
  Flexible PCB polyimide surfaces were used to integrate different potentiometric sensors^38^ with power management chips^39^ and temperature compensation modules^40,41^. Temperature compensation is often desired for electrochemical applications using enzymatic^40^ and not-enzymatic electrochemical sensors^41^. Flexible PCBs have also demonstrated the integration of surface mount (SMT) with electrochemical setups^42^, piezoelectric materials for SAW applications with IDT^43^ and incorporation of antenna setups with temperature/pH sensors^44^.
  Others have successfully fabricated microneedles^45^ on PCB substrates illustrating the seamless integration prospect of the PCB approach or fabricate PCB-based components with multiple functions. Hadi et al. fabricates IDEs on flexible PCB with microflow functions such as particle enrichment, active mixing and micro pumping with varying voltage applied^46^. Similar microflows using PCB-based electrodes have been achieved with optimized AC signal with specific frequency and amplitude used for simultaneous excitation of AC electrothermal (ACET) effect and interrogation of capacitance change at the electrode surface^47^.
- **Unconventional integration**
  Despite the lack of multiple components, or the inclusion of microfluidics, some works have demonstrated the wide range capabilities of PCB manufacturing when it comes to diagnostics by fabricating single-component electrochemical sensors of unique shapes. Toldra et al.^48^ have fabricated a PCB used for nucleic acid amplification by including the 3-electrode setup in conically shaped strip for the insertion of the point-of-care device in testing tubes (Fig. 3c) while others created USB-shaped PCB platform for seamless integration. USB shaped PCBs were used for the detection of HER2-positive breast cancer cells^49^ and microfluidic electrophoresis chips to separate proteins^50^. Even though there is no standard integration in these cases, the shape is rather unique, and intriguing, it showcases the ability of PCB based platforms for the full integration to not just sensors and electronics but for real-life and connectivity applications.
- **Commercially fabricated microfluidic layer**
  Perhaps, the highest importance and benefit of PCB technologies is the ability to seamlessly integrate microfluidics via the multilayer approach. Vasilakis et al.^51^ exploit the multilayer PCB design to fabricate a microfluidic active diluter with a variable and actively controlled dilution ratio (Fig. 3d), comprising a power MOSFET and digital temperature sensors, and an electrochemical biosensor. The device consisted of a 3-layer FR-4 stack, compromising a top, middle and bottom layer. The top layer was silver plated with the vias employed as pseudo references electrodes; middle layer was gold plated with the vias being exhibited as sensing electrodes. The microfluidics of the devices were realized using dry film photoresists through standard lithography on the bottom layer, which interconnects the inlet and outlet vias of the PCB. The authors, however, state the limitations of the device being the intrinsic hydrophobicity of the PCB manufacturing materials, which required the oxygen plasma treatment of the microfluidic layer to enable the filling of the device under pressure.
  The multilayer was also utilized to include cooling microchannels in PCB structures^52^. A four-layer FR-4 was created using prepreg sheets to avoid corrosion, creating microfluidic structures as the middle layer of the PCB stack. The water-cooling experiment was performed on the PCB with a plastic radio frequency (RF) power amplifier (PA), decreasing its temperature from 86 °C with a static fluid to 46 °C at a flow rate of 30 mL/min. In a similar manner^53^, a digital microfluidic device composed of a two-layer PCB was demonstrated to integrate a droplet ejection module with the droplet manipulation layer encompassing the PCB-based electrodes. The device was a double-layered DMF microchip with an oil-filled medium flipped over, with a liquid infusion port and a liquid expulsion port accommodated on the top working PCB plate and the bottom grounded ITO plate, respectively, to facilitate chip-to-world delivery of droplets. Similarly, Kim et al.^54^ utilizes the vias of the PCB layer, integrating acoustically oscillating bubbles for mixing to an electrowetting on-dielectric (EWOD) chip, which can generate microstreaming inside the droplet.
  Papamatthaiou et al.^55^, demonstrated for the first time the capabilities of mass manufacturing of the multilayer technique. For the first time, a custom-made Lab-on-PCB was designed in Altium® and was commercially, mass-manufactured in an established, standardized factory (£2.50 per chip) of dimensions 4.4 cm × 8 cm (Fig. 3a). It implements an embedded, double-channel layout (FR4 type 106, routed to create channel for fluids) for continuous flow operation incorporating the SARS-CoV-2 test with positive and negative controls. LoCKAmp is a complete diagnostic system centered on the core-Lab-on-PCB technology, seamlessly integrating continuous flow, miniaturized LAMP PCB chip with a simple, ultra-low-cost optical detection module and a seamlessly integrated thermal lysis module. The thermal lysis module is seamlessly integrated using the embedded resistive copper microheaters. Successful SARS-CoV2 detection and quantification in wastewater-derived RNA samples was demonstrated within 7 min with viral load information, or 2–3 min when a yes/no answer was required, at concentrations as low as 17 gc μL^−1^. The device tackles standardization challenges in lab-on-chip research and paves the way for the adoption of Lab-on-PCB technology for rapid laboratory-level molecular diagnostics outside a controlled laboratory setting, by far exceeding existing lateral flow tests in terms of time-to-result and performance^52^.

### Hybrid Lab-on-PCB integration

We are defining hybrid Lab-on-PCB as the separate design and fabrication of PCB-based substrates and their subsequent bonding to polymer microfluidic structure. As opposed to seamless devices, which require extensive design thought to consider layout and integration of the different components, hybrid devices are comparatively easier to design. Exploiting the already mature PCB and polymer fabrication technology, electronics and sensors can be easily integrated with microfluidics. The challenge for these devices often lies with the leak-free bonding of the fluidic components to the substrates. However, since fluidic and electronic chips are processed individually, full use of PCB and polymer fabrication techniques can be utilized with the only challenge being the suitable approach to combine the two.

**Fig. 4:**
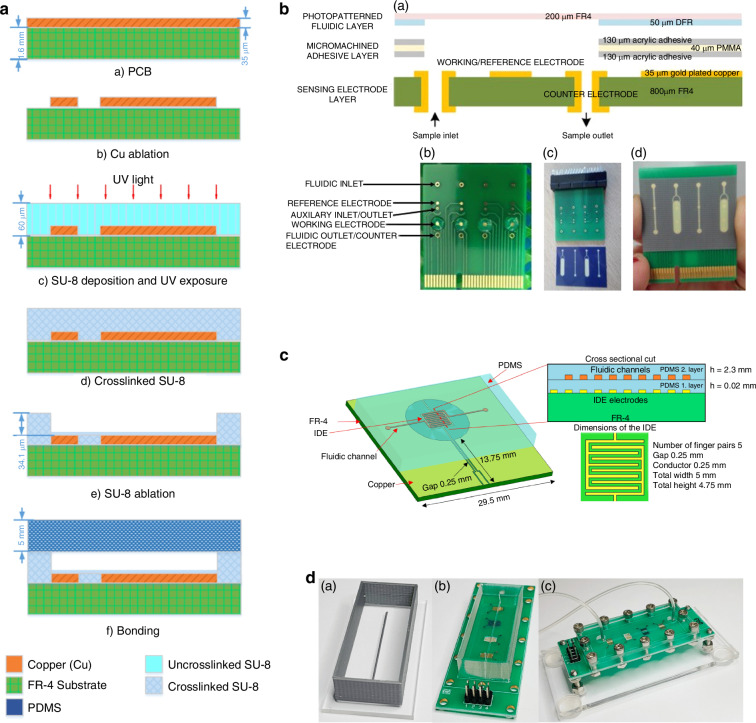
Hybrid Lab-on-PCB approaches. **a** Maskless fabrication of interdigitated electrodes and SU-8 microchannels on PCB substrate (reproduced from ref. ^58^, copyright 2021, with permission from IOP Publishing). **b** Lab-on-PCB electrochemical biosensing platform with adhered microfluidic layer, reproduced from ref. ^104^, copyright 2019, with permission from Elsevier. **c** PDMS microfluidic channel integrating a microwave sensor consisting of an interdigitated electrode (IDE) for detecting saline in biological range, reproduced from ref. ^66^, copyright 2019, with permission from MDPI. **d** PDMS channel fastened using mechanical screws aligned on four collinear sensing IDEs for an impedimetric e-tongue in a single microchannel application, reproduced from ref. ^130^, copyright 2020, with permission from MDPI

The integration of the fluidic component to the PCB layer often requires the modification of the electronic layer, as a flat surface is required to ensure leak-free bonding between the polymer chip and PCB. Extensive techniques were demonstrated, which can be defined according to the different fabrication methods utilized to bond microfluidics and electronics. We define two types of hybrid Lab-on-PCB devices, stacked-up and separate types. The stacking approach sequentially integrates electronic sensors and microfluidic components on flat, planarized substrates followed by a sealing layer to form a fully assembled device. The LoC-to-LoC approach works by individually fabricating polymer and PCB LoCs, with the bonding and assembly of the device at the end. Examples of the different structures are shown in Fig. 4.

- **Stacking approach**

The stacking approach was the first kind of hybrid structure, evolving from the seamless integration of lithography and etching-based structures. The microfluidics are fabricated on PCB substrates usually by depositing or spin-coating liquid polymers to planarize the surface and build channels.

#### Su8

Flores et al.^56^ fabricates SU-8 channels via lithography on top of PCB substrates. An impulsion system is created which includes microvalves that are connected to pressurized chambers. Once the microvalves are opened, the air is released impulsing the fluids. The microvalve is a copper fuse fabricated using the copper layer of the PCB. In addition, a SU-8 wall located over the fuse separates a pressurized chamber from the rest of the microfluidic circuit. The actuation of the microvalve implies the destruction of the fuse, opening the SU-8 wall.

Contreras-Saenz et al.^57,58^, by-passes the need for a mask for photolithography, and uses ablation to pattern the copper electrodes and SU-8 channels (Fig. 4a). Cu electrodes are fabricated by ablation, after which SU-8 is deposited to cover the whole surface of the PCB, and exposed and crosslinked under UV light. Then laser ablation is used to remove the unwanted SU-8 and form the microchannel before sealing the device with oxygen plasma bonded PDMS. The active microfluidic system was then applied for Electrochemical Impedance Spectroscopy (EIS) analysis performed at different Chinese Hamster Ovary (CHO) cell concentrations (105 to 108 cfu ml^−1^) to validate device assembly and functionality^58^. Although SU-8 combines well with the lithography technology, it requires high-end equipment, high cost and complicated fabrication process which limits large-scale manufacturing targets.

#### Dry film

As opposed to SU-8, dry film photoresist (DFR) which is also PCB compatible has better mass production possibilities. DFR comes at a lower cost offering better adhesion, uniformity and a better processing yield. Multiple studies have demonstrated the use of DFR in combination with PCB and applied the material for the fabrication of microfluidic-based PCB platforms for various applications. Vasilakis et al.^59^ used DFR to create PCB-based capillary pumps, while others create micropillar wicks for a microfluidic particle growth channel^60,61^. The DFR microfluidic structures were rendered hydrophilic under oxygen plasma and coating using surface graft polymerization, respectively. DFR was also used for actively driven microfluidics, with the use of sealing layers, patterning microfluids on FR-4 materials with embedded electrodes for isothermal recombinase polymerase amplification (RPA)^62^, DNA amplification^63^ and real-time analysis of size distribution and effective density of airborne nanoparticles (NPs)^64^.

#### Molds to pour PDMS

Although biocompatible, different polymers were also applied as a planarizing layer and polymer-to-polymer bonding for polymer intrinsic microfluidics countering the challenge of DFR bonding with polymers. PDMS is an example of polymer which was demonstrated as a planarized materials, encapsulating surface mount components and bonding microfluidic chambers to PCB substrates. The technique was used to integrate microfluidics with surface mount electroosmotic pumps^65^ and Optical components(CMOS) via milling the cured PDMS.

Constraining using molds to fix the PCB platforms, and then pouring PDMS to create a planar level and form microfluidics has been demonstrated for a microfluidic, IDE-based microwave (Fig. 4c) sensor detecting saline in biological range^66^, amperometric detection sensor of H_2_O_2_^67^ and the packaging of a QCM sensor on a microfluidic PCB^68^. Fixing wires^69^ and tubings^70^ at the bottom of molds before the pour of PDMS has also been demonstrated to quickly, and cost effectively construct microfluidic networks. The wires were then removed after the curing process leaving behind microfluidic geometries.

#### Hollowed out PCBs

A few studies have used the concept of hollowing out PCB, as a form of stacking up and integrating extrinsic components together. It relies on thin PDMS films on the bottom of the PCB to seal the hollowed-out area and form the cavity within the PCB before mounting sensors and microfluidic networks within the cavity. This method was demonstrated to include piezoelectric devices^71^, paper microfluidics^72^ surface acoustic wave sensors^72,73^ and active silicon chips, such as MEMS^74^.

- **LoC-to-LoC approach**

To remedy the defect of planarizing the surface, which involves extra steps in the fabrication process, separate Lab-on-PCB was introduced. This type of structure provides full use of the PCB and polymer fabrication process, enabling the bonding between extrinsic materials with its challenge being the final step, the full assembly and bonding of the separately fabricated modules.

#### Plasma

Low cost techniques have been utilized such oxygen plasma treatment, rendering the surface of polymers such as PDMS hydrophilic to enable its bonding to PCB or other polymer substrates.

Ying et al.^75^ bonds a PDMS microfluidic structure to a thin layer of PDMS on the PCB surface used for planarization using oxygen plasma treatment. After bonding, the chip could be used for the biophysical measurement of red blood cells. Using a similar process, others measure the electrical properties of HeLa cells on pre-patterned copper electrodes^76^ and sort cells using dielectrophoresis on PCB interdigitated electrodes^77^. Separate structures however, don’t only allow the bonding of polymer to polymer, with multiple studies bonding PDMS components to SU8^78^, PMMA to PDMS^79^ PDMS to metal^80^ using plasma and APTES crosslinking and PCB surfaces^48,81–86^.

The microfluidic structures are fabricated using soft lithography, with molding techniques with the mold fabricated using 3D printing and lithography techniques.

#### Adhesives

Another simple but effective procedure to integrate microfluidics and PCBs is the use of adhesives, often in the form of double-sided tapes or glue. Tape adhesives were used to adhere PDMS microfluidic structures on PCBs for applications such as resonators for microwave frequencies^86^, broadband and polarization-insensitive absorption^87^, NFC antennas^88^ embedded cooling structures^89^ electrochemical applications^90–92^ biological applications^93^

Similarly, PMMA blocks were boned for microchip electrophoresis^94^ electrochemical applications^95–97^ amplification techniques^98,99^ electrolytic micropumps^100^

The adhesive layer can also be utilized as the spacer to create microfluidic chambers, as demonstrated by Zhang et al. for the fabrication of a microfluidic device for ion sensing^97^, Ji-Soo^101^ for a DNA amplification device and Huang et al. for electrochemical detection of uric acid using a silicone spacer and coverslip as sealing^102^ and Delianides et al.^103^ for a Multichannel Miniaturized Dielectric Blood Coagulometer.

Jolly et al.^104^ present a microfluidic stuck compromising a thin FR4 layer (to allow for the fluidic optical inspection), laminated with a 40 µm thick DFR (Fig. 4b). The resist is patterned via conventional photolithography and developed. A custom adhesive layer was employed to form the final stack, laminating a 50 µm thick PMMA film with 3M 468MP acrylic adhesive; the adhesive layer was laser micromachined following the fluidic layer pattern and then pressed at room temperature between the fluidic layer and sensing layer to achieve leak-tight sample flow. Dutta follows this technique for an enzyme-assisted glucose patch^105^

Laser micromachined microfluidics and adhesive bonding were also demonstrated for inkjet-printed pseudo reference electrodes for lab-on-chip integrated electrochemical biosensors^106^, development of microfluidic Y-channel^107^ while another study^108^ uses three thin layers of flexible patterned polyethylene terephthalate (PET) to integrate a bacteria-responsive DNA hydrogel-coated gate electrochemical transistor.

Similarly to tape adhesives, glue adhesives can also be utilized to hybrid-like integrate microfluidics to PCB for several applications such as interdigitated electrode (IDE) for Point-of-Care Detection of SARS-CoV-2^109^, biological applications^110^, pressure sensors^111^ and microfluidic impulsion systems^112^

Adhesives can also provide the bonding of 3D printed microchannels^113^ and paper microfluidics^114^ to PCB substrates, materials which don’t tend to bond using previously discussed plasma and surface treatment techniques.

#### Half dry

Researchers used PDMS as an adhesive using the “half-dry technique”^115–117^ where the PDMS is baked until it is nearly complete dry before putting the complete dry microfluidic layer on the surface of the “half-dry” PDMS. Finally, both layers are thoroughly baked until dry for their tight combination to create the assembled device. Others applied thin layers of PDMS to planarize the surface and provide an adhesive surface for PDMS^117–122^ to bond to the PCB substrate. This technique was used for applications where adhesion is not feasible between the surface (such as metal) and polymer and to avoid the use of non-biocompatible adhesives.

#### Mechanical fixing

It is often used as cost effective, and simple method for the packaging of microfluidics with PCB platforms. Resin 3D-printed microfluidic chambers were attached to PCB platforms via the means of screws and casing structures combining ISFETS and pseudo reference electrodes^123^ and Integrated Electrochemical Quartz Crystal Microbalance (IEQCM)^124^ with microfluidic layers. 3D casings were also used to introduce paper microfluidics on PCB surfaces^125^. Other sandwich microfluidic blocks and PCBs under mechanical pressure using plates and screws include applications for acoustic pumps^126^, EGFET glucose sensing^127^, biofuels^128,129^ electronic tongues (Fig. 4d)^130,131^ DNA detection^131,132^, gel preparation^133^, dielectrophoresis^129,134^, fluid manipulation^135,136^ and capacitive^136^ and impedance sensing^132^. Figure 4d depicts a robust e-tongue device comprising all sensing units in a single microchannel allowing automated multiplexing of the sensor array, used to investigate the Influence of the Flow Rate for Sucralose Differentiation.

#### Unconventional techniques

Franco et al.^137^ demonstrate an interesting technique integrating a PMMA microfluidic chip on a PCB by exploiting the on-chip microheater for the melting of PMMA. The microchannels are micromachined in a piece of PMMA using a CNC milling machine, and PCB is done using a typical chemical wet etching technique. Vias are drilled to the PCB and PMMA piece to allow air flow from the internal region to the outside, when the piece of PMMA is placed over the PCB. Finally, both parts are aligned and the microheaters are connected to a power supply to provide a constant value of current. At the same time, a pressure of 6.3 kPa is applied on top of thermoplastic part ensuring the melting of the PMMA and its bonding on the PCB. The same technique was used at a later stage to create a highly integrable and normally open microvalve for industrial thermoplastic-based lab on PCB^138^.

Another technique bonding PMMA to PCB, uses solvent for the surface modification of PMMA, bypassing the use of plasma. One study^134^ investigates the use of different solvents and their ability to provide leak-free bonding of PMMA to PCB substates. The PMMA laser engraved microfluidic structure was bonded to PCB by exposing the interface surfaces to solvent at room temperature and then applying moderate analytical grade chloroform, ethanol and acetone. While the result was better for chloroform, the overall bonding quality was not good. A combination of plasma treatment and ethanol coating was demonstrated for bonding of PMMA for a continuous flow µPCR chip for DNA amplification^139^.

### Modular Lab-on-PCB

A prospective approach to design and fabricate Lab-on-PCB is the modular approach. Modularity in PCB design allows the development and design of separate modules/components, which can then be integrated and be combined depending on the application. Advantages include the flexibility of combining different components for specific applications and the full use of the fabrication techniques when considering individual, simpler components than a whole integrated system.

Modularity has been demonstrated in LoC before, with the focus being modular microfluidic devices. Modular microfluidics can be fabricated by normal means, such as soft lithography, 3D printing, and pressure sealing approaches^140–144^. Modular microfluidics can be fabricated by normal means, such as soft lithography, 3D printing, and pressure sealing approaches^140,142–144^.

Microfluidic ‘pieces’ molded using SU-8 with integrated tongue and groove connecting features have been developed^140,145^ while others have based their approach after LEGO, creating components via 3D printing^145^ or micromachining^140^, relying on the hydrophobicity of PDMS, O-ring and tubing for leak-free integration assembly between ‘pieces’. In addition to the nesting of physical structures, modular microfluidics have successfully been configured using magnetic-aided connections^143^. Modules have been fabricated via normal means such as 3D printing with embedded ring magnets in their inlet and outlet ports^146^. The magnets were then confined with sealing gasket techniques such as square profile O-rings or Kapton polyimide adhesive tapes with center holes. Connecting the different modules can then be achieved by stacking up modules of ports of opposite magnet polarities. A deliberated design is needed regarding the channel design, as it must match the profile and size of the magnet used. Generally, modular techniques require highly evaluated thought prior to the design of the different modules, to achieve leak free sealing and integration.

Unlike LoC, Lab-on-PCB can be used to achieve modularity between electronic components too and not just between electronics and microfluidics. Furthermore, the integration of sensors can be achieved easier on the PCB substrate rather than encapsulating different sensors in Polymer chips.

**Fig. 5:**
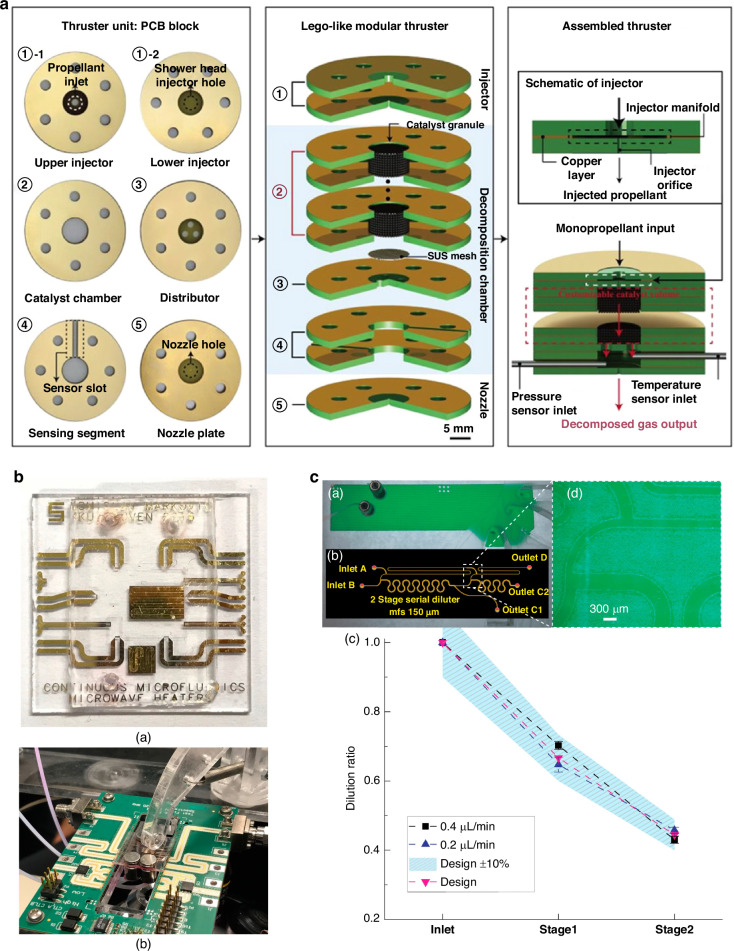
Modular Lab-on-PCB approaches. **a** PCB blocks used for modular monopropellant thruster fabrication, reproduced from ref. ^153^, copyright 2024, with permission from Elsevier. **b** Microwave-microfluidic chip placed on top of the microwave-microfluidic PCB platform and fixed by the spring-loaded plastic pin used for thermal actuation of nanoliter volumes in continuous microfluidic (CMF) channels, reproduced from ref. ^147^, copyright 2019, with permission from MDPI. **c** Cascade format of a PCB based two-staged diluter, reproduced from ref. ^144^, copyright 2019, with permission from MDPI
- **Cascade**
  Similarly to LoC, microfluidic network blocks/units have been demonstrated on commercial PCB substrates for dilution uses. Microfluidic networks of modular pressure and flow rate were fabricated on standard FR4 sheets and a 64 μm thick DFR, patterned using standard photolithography^144^. The final PCB device compromised of the PCB-based microfluidics at the bottom, a sealing PET layer and two additional, adhesively bonded PMMA layers for microfluidic interfacing. The microfluidic networks were designed on a cascade geometry PCB layer, with the ability to merge two or more devices during the sealing process (Fig. 5c). The modular unit cell is compatible with multistage serial dilution network applications. Adopting a modular PCB design, using physically separate boards for control logic and electrodes connected by ribbon cables, Eswar et al.^148^ presents an automated digital microfluidic device for dairy milk droplet actuation.
- **Lego-like microthruster**

- **Spring loaded**
  Markovic et al.^147^ designed a continuous flow microwave-microfluidic chip, based on an Interdigitated Capacitor design (Fig. 5b). The device can sense and locally heat nanolitre volume at specific locations in the fluid channel, which is established using PDMS. The chip integrates an IDC for heating, platinum resistors for accurate local contact-based temperature measurements using a digital mustimeter and meander geometries for mixing and cooling zones. The device is integrated using a spring-loaded plastic pin on a microwave PCB, which allows electrical access to the heaters and platinum resistors for the local temperature measurements and microwave excitation of the IDC. The PCB platform allowed simultaneous fluidic actuation and optical inspection of fluid flow through an opening in the PCB.

In a similar manner to LoC, where microfluidic blocks were assembled in a LEGO manner^140,145^, an innovative modular fabrication method for a green monopropellant thruster, employing PCB as fundamental building blocks for rapid and efficient performance optimization was demonstrated^149–152^. Employing the PCB multilayer approach, thermal and structural characteristics are superior to conventional MEMS-based microthruster. The PCB blocks consisted of unit and functional blocks manufactured commercially using circular-shaped FR-4 substrates^153^. These functional blocks serve as the building blocks for the various components of a thruster (Fig. 5a), which when assembled components such as injector, decomposition chamber, catalyst chamber, and nozzle can be constructed. The injector block included an upper and lower injector, the decomposition chambers, a catalyst chamber, distributor, and sensing segment (a block for inserting sensors for chamber monitor) and the nozzle is composed of a single component. The final device was then assembled using mechanical fixing through drilled holes on the different layers.

The PCB industry has demonstrated modular integration for decades, a development direction which if followed and adopted, modular microfluidics could support the standardization and mass production of Lab-on-PCBs in the future.

### Comparison of Lab-on-PCB and other upscaling technologies

Lab-on-PCB primarily uses fiberglass-reinforced epoxy laminates (FR-4) commonly used in the PCB industry, as opposed to mainstream semiconductor manufacturing materials used in LoC, such as silicon and glass. Both technologies can achieve integration of microfluidics using polymer layers, such as PDMS, machining or via photolithographic techniques. Interestingly, Lab-on-PCB does not necessarily require these integration techniques, as previously discussed in Section 2, the microfluidic integration can be achieved seamlessly by exploiting multilayer PCB technologies, the commercial PCB fabrication advances and 3D printing procedures for integrating microfluidics on PCB substrates. Manufacturing technologies, such as photolithography and surface modifications or bonding techniques which rely on cleanroom conditions, can be utilized by both technologies, with the key difference being in the achievable minimum feature size. Unlike LoC, PCB manufacturing does not require necessarily semiconductor cleanroom conditions, reducing the costs for fabrication, at the same time diversifying the manufacturing options geographically. Reducing the cost of materials and production, providing integration with electronics, a better prospect of upscaling due to standardization in PCB production, Lab-on-PCB addresses the limitations observed in the LoC technology. Table 1 provides a comparative analysis of various LoC technologies—Paper, Glass, Silicon, and Lab-on-PCB—outlining their respective advantages and limitations about cost, fabrication complexity, integration potential, performance and ideal application scenarios.

**Table 1:**
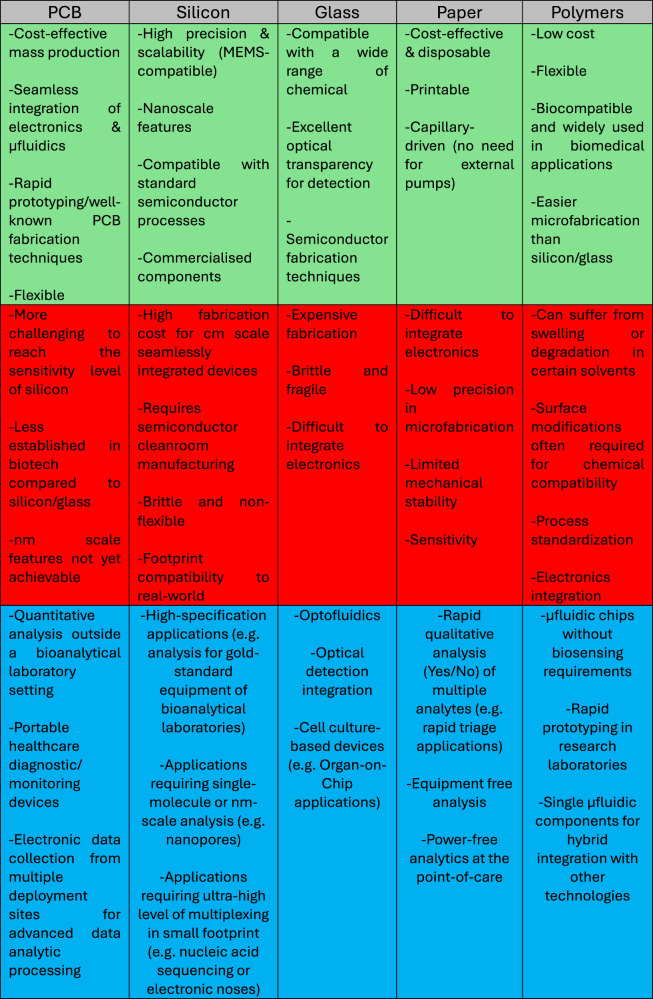
Comparative table of Lab-on-PCB and other μTAS technologies (green: advantages, red: drawbacks, blue: ideal application)

## Applications of Lab-on-PCB

### Fluid handling and manipulation

Microfluidic technology has driven the development of Lab-on-PCB systems, allowing for efficient fluid handling and manipulation across applications in environmental monitoring, chemistry, and biology^22^. This subsection critically reviews recent advances in Lab-on-PCB-based fluid handling methods, including electrowetting on dielectric (EWOD) and dielectrophoresis (DEP). Each of these approaches enables precise droplet control in compact, low-cost systems suitable for varied microfluidic applications.

Digital microfluidics (DMF) has emerged as a prominent method for droplet manipulation within Lab-on-PCB, primarily leveraging EWOD technology^54,150–152,154–170^. By applying electric fields, EWOD devices alter the wetting properties of droplets, enabling control over droplet movement, merging, splitting, and dispensing without external pumps or mixers^22^. This mechanism consists of a hydrophobic electrode layer insulated from the droplet, allowing controlled actuation of small droplets (micro- to pico-litre scale) across an array of electrodes. DMF-based systems offer high throughput, minimal cross-contamination, and precise droplet control, positioning them as powerful tools for applications such as multiplex detection in micro- and nano-scale assays^154^.

For example, Li et al.^155^ introduced a novel DMF-based PCB platform for blood coagulation assays, facilitating dual-parameter testing for clotting tendency and clot stiffness. As coagulation occurs, blood droplet movement slows down across the electrode arrays. However, while dual-parameter coagulation analysis was achieved, it requires a high operating voltage (230 V), which may limit portability and compact integration, a common challenge across EWOD-based Lab-on-PCB systems^156,157^. Another significant contribution by Eswar et al.^148^ involved an on-site DMF dairy testing device for milk analysis. By reducing surface roughness with thin film parafilm dielectrics and adjusting EWOD force through direct current (DC) and alternating current (AC) modulation, the team minimized biofouling and improved droplet control in open configurations. These studies show the ongoing efforts to balance actuation efficiency with portability and system stability for real-world applications.

Several other studies have examined electrode design and material choices to optimize droplet movement. For instance, Zulkepli et al.^158^ observed that zigzag electrodes improved droplet velocity over square designs, while Al-Mogahed^156^ showed that PTFE-coated copper-tin electrodes increased droplet stability on Lab-on-PCB platforms. Other electrode patterns, including interdigitated and crescent shapes, achieved varied velocities and actuation voltages, demonstrating the sensitivity of droplet movement to electrode shape and surface properties.

**Fig. 6:**
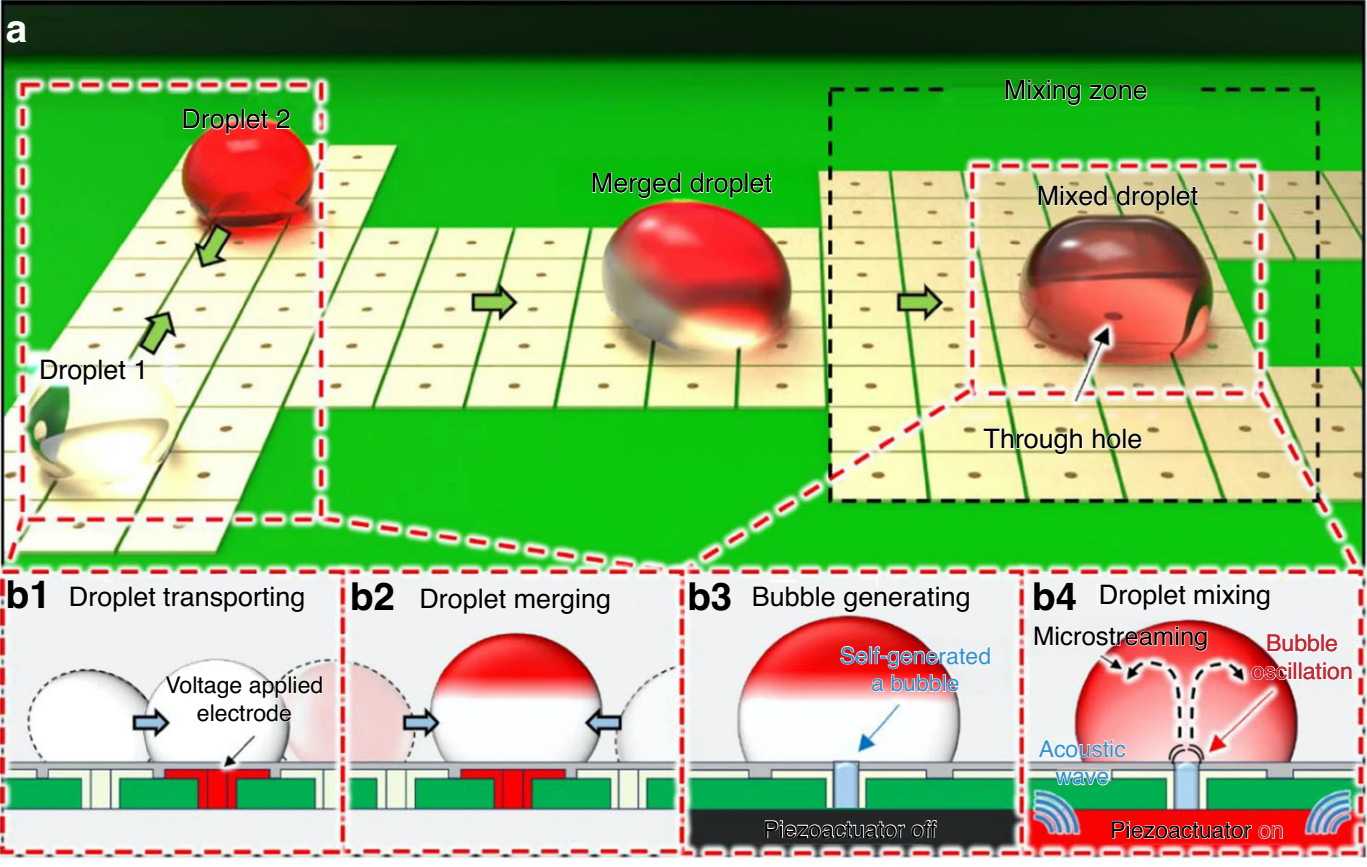
Schematic diagram of droplet manipulation process on the EWOD chip. **a** The DMF platform consists of an EWOD chip, driver board, and software. **b1** and **b2** show the transportation to the center of two different biochemical droplets. Then, they are merged by EWOD actuation on the chip. **b3** Once the merged droplet arrives at the mixing zone and positioned on the hole, the through-hole is used to naturally trap a bubble. An acoustic excitation with the resonant frequency of the bubble is applied, resulting in bubble oscillation and microstreaming. Finally, mixing within the droplet is enhanced, as shown in **b4**. Reproduced from ref. ^54^, copyright 2024, with permission from Springer Nature

Many Lab-on-PCB devices focus on droplet transportation and mixing. Diaz et al.^152^ utilized an EWOD biosensor to mix temperature-sensitive droplets for human papillomavirus (HPV) detection, demonstrating the potential of Lab-on-PCB for complex biochemical assays. Figure 6 shows an exemplary system of an innovative PCB-based EWOD chip, introduced by Kim et al.^54^, that uses an oscillating bubble in through-hole structures to generate microstreaming for droplet mixing. This approach achieved droplet velocities of 2 mm/s at 100 V_dc_ and 40 mm/s at 300 V_dc_, representing a novel strategy for enhancing mixing efficiency in compact designs. A detailed explanation of the working system is described in the caption of Fig. 6. Such variations highlight the diverse methodologies under development to enhance droplet mixing precision and control within Lab-on-PCB platforms.

Although EWOD remains a primary actuation mechanism, some studies have explored magnetic methods for droplet manipulation. Yen et al.^159^ designed a magnetically actuated Lab-on-PCB device utilizing a two-layer 4 × 4 micro-coil array to move droplets containing magnetic beads without micropumps, reducing power requirements. A similar magnetic approach, using clustered magnetic beads within Lab-on-PCB systems, was reported by Lin et al.^171^. These studies indicate that magnetic actuation can offer viable alternatives to EWOD, particularly if high voltages are not desired.

These findings highlight key limitations in DMF device fabrication, particularly regarding scalability and parallel multiplexed handling, which Ecken et al.^160^ aim to address. The team developed VAPE-DMF (Vertical Addressing of 1-Plane Electrodes for Digital Microfluidics), which separates Lab-on-PCB devices into two components: a closely spaced driving electrode cover and a sub-substrate for vertical addressing. This setup demonstrated robust handling of 48 droplets across 336 electrodes, showing promise for scalable, low-cost DMF systems with high multiplexing potential.

Some research has further examined alternative actuation methods to enhance fluid handling efficiency. For instance, Datta et al.^161^ provided an in-depth analysis of electrostatic actuation, including EWOD and DEP, suggesting a low-cost manufacturing approach outside cleanroom facilities. Another innovative approach by Wikramanayake et al.^150^ demonstrated electrowetting in microgravity, marking the first reported space-based application of Lab-on-PCB fluid manipulation.

Several Lab-on-PCB systems have incorporated droplet tracking, ejection, and separation capabilities for varied applications. In one study, EWOD capacitance sensing allowed simultaneous tracking of multiple droplets, enabling real-time monitoring in an open system with PDMS dielectric^162^. Optical monitoring has also been leveraged to track droplet parameters in Lab-on-PCB devices through open-source computer vision^163^ These advancements in droplet tracking enhance the potential for fully automated Lab-on-PCB systems, key for high-throughput, real-time assays.

For droplet ejection, studies have shown that adjusting electrode shape and layout can significantly influence dispensed droplet size. Ahmadi et al.^164^ reported that channel electrode curvature improved droplet release precision, while another study introduced a double-layered DMF microchip for automated droplet ejection, facilitating efficient chip-to-world transfer^53^.

**Fig. 7:**
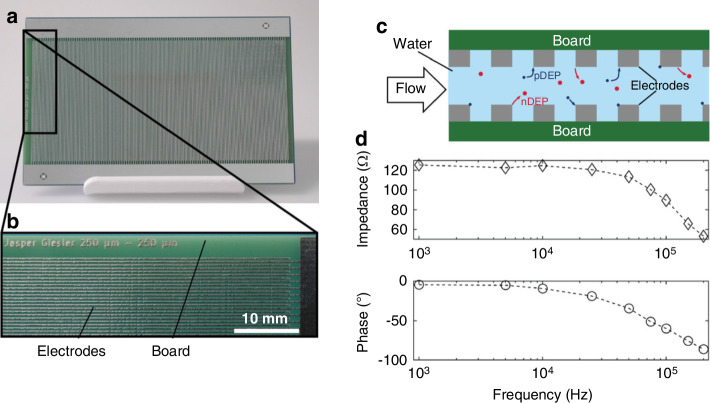
High-throughput dielectrophoretic seperator based on PCB. **a** Front view of the electrode array: The PCB measures 80 ×120 mm. The non-conductive substrate, shown in green, is made of FR-4 material, while the electrodes are composed of copper. A lead-free hot air solder levelling surface finish, in silver, is applied to the electrodes. **b** Close-up of the PCB: The electrodes feature a width and spacing of 250 µm each. **c** Schematic of the device: The design incorporates PCBs as the top and bottom layers, creating a channel through which a particle suspension flows. The electrodes produce an electric field, inducing positive (pDEP) and negative dielectrophoresis (nDEP) in the suspended particles. A silicone gasket (not depicted) separates the two boards. **d** Measured impedance and phase shift: During testing, water with a conductivity of 2.1 µS/cm was pumped through the channel, and the impedance and phase shift of the device were recorded. Reproduced from ref. ^172^, copyright 2022, with permission from John Wiley and Sons

DEP has also been employed for particle separation based on size, shape, and material properties, a critical step in biological and environmental assays. For instance, a high-throughput DEP separator designed with interdigitated electrode arrays demonstrated effective separation of polystyrene particles ranging from 500 nm to several micrometers^172^. As illustrated in Fig. 7, this DEP separator consists of a PCB-based electrode array that generates the necessary electric fields for particle manipulation. The figure highlights both the structural design of the device and its impedance measurements, demonstrating its effectiveness in DEP-based separation. Further studies have applied DEP for biological samples, such as blood cell separation^80^, cell separation^77^, and microorganism capture^173^, emphasizing DEP’s versatility in Lab-on-PCB applications.

In summary, Lab-on-PCB systems for fluid handling and manipulation have shown strong potential across applications in diagnostics, environmental monitoring, and biological research. Although DMF and EWOD remain the primary actuation mechanisms, challenges related to high power demands, electrode design, and droplet tracking continue to limit scalability and portability. Innovative approaches, such as VAPE-DMF for scalable electrode integration, magnetic, and DEP-based alternatives, are opening new avenues to address these limitations. Future research should focus on reducing power consumption, enhancing droplet control, and expanding application versatility to fulfill the commercial and practical potential of Lab-on-PCB technology.

### Lab-on-PCB for biological applications

In recent years, the application of Lab-on-PCB technology for biological diagnostics and biomarker detection has expanded rapidly. This growth likely stems from the capability of Lab-on-PCB to address limitations of traditional diagnostic devices, which are typically expensive, bulky, and time-consuming, making them less suitable for on-site testing and rapid response^21^. By integrating biosensors with PCB technology, Lab-on-PCB offers a streamlined, cost-effective platform for various applications, including cell analysis, immunosensors and immunoassays, biochemical assays, and disease diagnostics^21^.

One of the notable Lab-on-PCB applications in cell analysis is cytometry, where cellular properties such as size, count, and cell cycle are assessed. This is critical for understanding immune function, cancer progression, and other physiological states^21,174^. For example, a study by Fu et al.^85^ introduced an impedance-based microfluidic cytometer on PCB for differentiating and enumerating white blood cells and circulating tumor cells (CTCs) from red blood cells. Here, the disposable device leverages PCB electrodes to detect cell impedance variations in a microchannel, enabling label-free, on-site cell enumeration. Another similar device^93^ applies impedance to count cells and microbeads, demonstrating the flexibility of Lab-on-PCB for diverse particle analysis. Additionally, recent work^81^ demonstrated impedance flow cytometry for bacterial and microparticle analysis using square-wave excitation voltages across a PDMS-bonded microchannel, further underscoring Lab-on-PCB’s adaptability for microbial and cellular diagnostics.

**Fig. 8:**
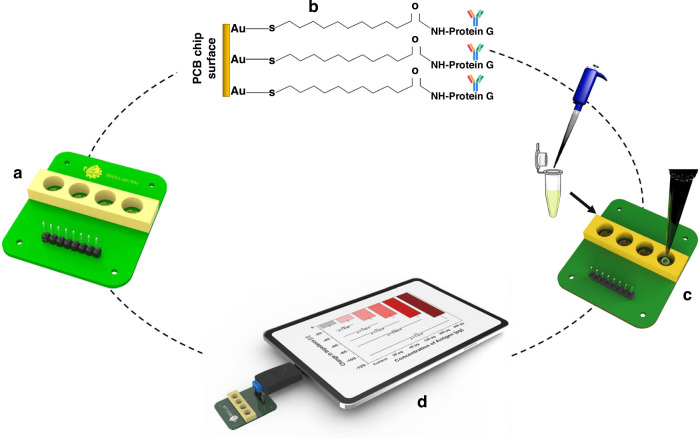
Schematic of Lab-on-PCB impedance-based sensing of viral NC and spike protein in nasopharyngeal swabs. **a** PCB used for viral protein detection. **b** MUA–Protein-G bioconjugation for antibody immobilization (Anti-SARS-CoV-2 NC/Anti-SARS-CoV-2 S) on the PCB. **c** Analysis of nasopharyngeal swabs from SARS-CoV-2 patients. **d** Microchip linked to a smart system for data acquisition. Reproduced from ref. ^109^, copyright 2022, with permission from IEEE

Lab-on-PCB platforms have significantly advanced immunoassays and immunosensors, key diagnostic tools in detecting infectious diseases^109^. Immunoassays typically use labeled detection methods, while immunosensors often provide label-free detection, such as electrical signal changes, allowing each approach to offer unique diagnostic. The COVID-19 pandemic, for example, accelerated Lab-on-PCB innovations in both areas, especially for detecting SARS-CoV-2. This surge in Lab-on-PCB research highlights the technology’s potential for rapid, low-cost diagnostics^55,131^. Notably, Rudge et al.^131^ developed a Lab-on-PCB-based bead immunoassay with electronic readout, eliminating the need for optical devices. By using enzymatic metallization, this system produces distinct impedance signatures, allowing electronic detection of SARS-CoV-2 antibodies directly in serum samples. Similarly, Joshi et al.^109^ designed a label-free Lab-on-PCB immunosensor of SARS-CoV-2 nucleocapsid and spike antigens with interdigitated electrodes, illustrated in Fig. 8, utilizing a biofunctionalization strategy that immobilizes antibodies on a PCB-based microwell to simultaneously detect nucleocapsid and spike proteins. This approach is credited with enabling a sensitive, robust, and reproducible electrical response to the target viral proteins. The authors emphasize the device’s operational simplicity, positioning it as a cost-effective, high-throughput point-of-care (POC) system suitable for reliable SARS-CoV-2 screening.

**Fig. 9:**
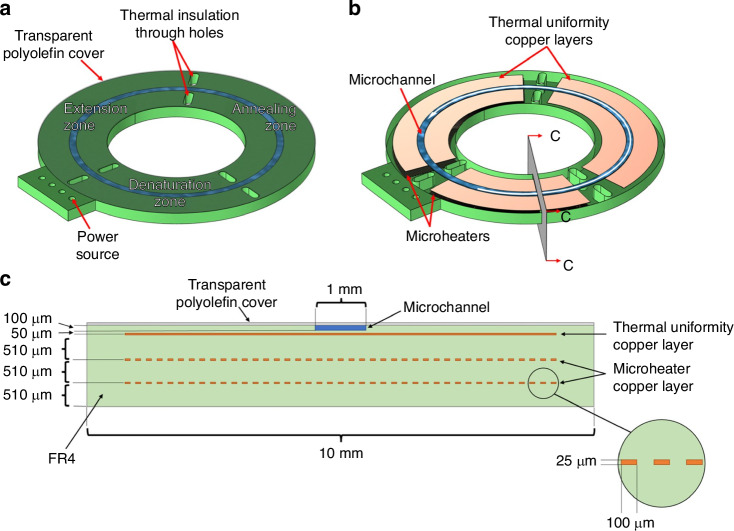
Geometry of the CL Microreactor. **a** External view: The microreactor consists of a 40 mm diameter PCB chip with a central 20 mm hole. A circular microchannel, covered by a thin polyolefin film, is divided into three temperature zones for PCR steps. **b** Internal view: The sample flows counterclockwise (white arrow) through the microchannel (100 μm deep, 1 mm wide, 94.25 mm long) across the three PCR temperature zones. Copper microheaters (25 μm thick, 100 μm wide) embedded in the PCB regulate the denaturation and extension zones, while the annealing zone cools passively. Elliptical through-holes between zones enhance thermal insulation. **c** Cross-section (C–C plane): The cross-sectional view highlights the integrated microheaters and copper layers, ensuring thermal uniformity across the microreactor. Reproduced from ref. ^176^, copyright 2023, with permission from MDPI

Other than immunosensors and immunoassays, Lab-on-PCB devices are expanding into nucleic acid amplification tests (NAATs), delivering significant improvements in rapid, sensitive pathogen detection, and positioning Lab-on-PCB as a pivotal tool in modern diagnostics^63,99,139,175^. NAATs on Lab-on-PCB, particularly polymerase chain reaction (PCR)-based approaches, exemplify how miniaturization on PCB can bring laboratory-level precision directly to the point of care. For example, Skaltsounis et al.^176^ developed a closed-loop microPCR on PCB as illustrated in Fig. 9, which depicts the geometry and structural design of the device. The microPCR system features a circular microchannel that allows continuous sample flow through three distinct temperature zones: denaturation, annealing, and extension. This was optimized for rapid thermal cycling. The integration of microheaters and thermal insulation layers ensures efficient heat distribution, enabling the system to complete 30 PCR cycles in under three minutes. This compact, low-power design offers major advantages for point-of-care diagnostics, with potential future enhancements focused on integrating real-time detection capabilities.

Beyond PCR, the adaptability of Lab-on-PCB extends to other NAAT methods, including loop-mediated isothermal amplification (LAMP)^55,98,177^ recombinase polymerase amplification (RPA)^48,62^, CRISPR-based assays^178^, helicase-dependent amplification (HDA)^32^ and isothermal strand displacement amplification (iSDA)^98^. These alternative methods allow Lab-on-PCB platforms to avoid the complex thermal cycling required by PCR, making them especially suitable for low-resource settings. For instance, Papamatthaiou et al. pioneered the first commercially manufactured LAMP-based Lab-on-PCB system for SARS-CoV-2 detection, employing continuous-flow fluorescent detection in a low-cost, compact format that offers rapid, high-sensitivity genetic testing^55^. This advance illustrates Lab-on-PCB’s potential to meet urgent testing demands with rapid, high-sensitivity genetic testing that is feasible even in resource-limited settings. Moreover, recent innovations by Shah et al., who developed a USB-powered Lab-on-PCB for isothermal NAATs (iSDA and LAMP) with on-board heating for pathogen lysis and amplification, underscore Lab-on-PCB’s potential for fully integrated, plug-and-play pathogen testing^98^. Such designs highlight the flexibility and adaptability of Lab-on-PCB platforms, demonstrating their potential as field-ready solutions.

Electrochemical Lab-on-PCB-based devices are also applied in diverse biological contexts beyond NAATs. Applications include protein analysis^50^, cell property characterization^76^, and DNA detection^91^. For example, field-effect transistor (FET)-based sensing can detect biomolecular interactions with high sensitivity, an essential feature for monitoring infectious agents and other biological analytes^123^. For instance, Papamatthaiou et al. developed an electrolyte-gated FET on Lab-on-PCB with graphene ink drop-casting, tailored for PNA probe attachment on the graphene channel to enable label-free DNA detection, particularly targeting oncogenic PIK3CA mutations. This setup, using in-plane PCB electrodes, facilitates miniaturization and integrated bio-sensing without the need for external reference electrodes^36^.

Different Lab-on-PCB detection methods other than electrochemical have shown potential in a range of applications, including clinical and biological applications. Capacitive sensors^47,125,147,179,180^ for example, have been used to detect virus-infected cells by monitoring capacitance variations, offering rapid and early detection without the need for complex sample preparation^181^. SAW sensors^43,73,82^ have proven effective for measuring physical and chemical properties such as fluids and viscosity measurements. These broader applications show Lab-on-PCB technology’s rapid expanding role yet emphasizing the need for advances in performance standardization to meet the demands of real-world applications.

### Sensing and detection

Recent advances in Lab-on-PCB-based devices have highlighted electrochemical sensing as a primary focus, where analyte reactions generate detectable electrical signals^22^ Electrochemical approaches enable efficient detection of various electrolytes and biomarkers, critical for diagnosing and monitoring health conditions such as dehydration, cardiac output, and neurological disorders^182^. This subsection evaluates recent Lab-on-PCB-based electrochemical sensing advancements, focusing on electrolyte monitoring and biomarker detection, with an emphasis on their potential for scalability and practical use.

Electrochemical Lab-on-PCB devices have shown potential in detecting physiologically important electrolytes like sodium and potassium. For instance, Salerno et al.^37^ developed a scalable Lab-on-PCB platform utilizing electrolyte-gated graphene field-effect transistors (EG-GFETs) and sprayable graphene ink to detect pH and Na+ in aqueous solutions. This system achieves high pH sensitivity (143 ± 4 μA per pH unit) and a linear Na+ response, highlighting its viability for commercial sensor arrays. Similar electrochemical platforms have achieved promising results in detecting trace mercury^114^ potassium^38,113^ sodium^38,183^ and pH levels^37,184,179^.

Lab-on-PCB platforms have also shown significant promise in biomarker detection, providing early diagnostics for diseases that often develop asymptomatically, such as cancer^185^. Electrochemical biosensors are widely used for this purpose, with recent studies demonstrating the sensitive detection of multiple biomarkers (Table 2).

**Table 2:** Detection of different biomarkers using electrochemical methods

PCB Electrode Modification	Biomarker	Limit of Detection (LOD)	Level of Integration	Refs.
Methylene blue (MB)	Myeloperoxidase (MPO)	15.79 ng/mL	Medium	^90^
Au-decorated boron nitride nanotubes	Metalloproteinase 9	0.0116 ng/mL (in Buffer) 0.0218 ng/mL (in Serum)	Low	^213^
Lactate oxidase	Lactate	-	Low	^214^
ZnO nanorods (NRs) and ZnO NRs: reduced graphene oxide (RGO) composites	8-hydroxy-2′-deoxyguanosine	100 fg/mL	Low	^215^
Nafion/Tyrosinase/RuO_2_/ENIG	Dopamine	0.12 µM	Low	^216^
ZnO nanostructures immobilized with ZIKV-NS1 antibody	ZIKV-NS1	lower than 1.00 pg/mL	Low	^217^
DNA 3 way-junction /Au nanospike	Troponin I	24 pg/mL (1pM) (in a dilluted serum)	High	^186^
MoO_3_	Acetylcholine	8.72 nM	Low	^218^
Thiolated (-SH) antibody	Streptococcus mutans bacteria	10^3 CFU/mL	Low	^219^
Apt_CA-125_/AuNPs@ nitrogen-doped mesoporous carbon nanomaterial /Au circular plated electrodes Array	Carbohydrate antigen 125	0.1-300 U/ml	Low	^220^
Thiolated single-stranded DNA (ssDNA) probe	SARS-CoV-2	0.50 μM	Low	^221^
Graphene Streptavidin Biotinylated ssDNA	synthetic DNA strand matching the sequence of ORF1ab	100 fg/mL	Medium	^222^
Thiolated ssDNA/Au	Salmonella typhimurium	7.6 nM	High	^223^
Methylene Blue (MB)	Tobramycin	125 µM	Low	^224^
Nafion/Uricase/ZnO	Uric acid	7.14 μM	Low	^179^
RuO_2_ sensing film	Urea	NA	High	^39^
Organic electrolyte gated FETs, and Molecularly Imprinted Polymer	Cortisol and 8-isoprostane	NA	High	^79^
Creatinine deiminase/Polyaniline Ni-Nafion	Creatinine	2 µM	Medium	^96^
Label-free line-pad-line/DNA aptamer	Vascular endothelial growth factor (VEGF_165_)	0.017 fM	Low	^225^

Level of integration considers the number of components integrated on the PCB platform. Low consider singular component PCB-based biosensors, medium integration of microfluidics with singular PCB based biosensor and high when there are three or more components, fluidic or electronic

Lee et al.^186^ introduce a promising Lab-on-PCB biosensor for label-free cardiac troponin I (cTnI) detection, crucial for acute myocardial infarction (AMI), also known as heart attack, diagnosis. By incorporating a DNA three-way junction (3WJ) on gold nanospikes (AuNS) integrated with a PCB micro-gap electrode, the device achieves notable sensitivity, with a detection limit of 1.0 pM in buffer and diluted serum. This design offers flexibility and potential for integration with accessible, portable platforms such as Arduino or Raspberry Pi. However, while these results are promising, further studies are required to confirm the sensor’s robustness in real-world conditions, especially given the complexity of clinical samples.

Nandeshwar et al.^90^ addresses common limitations of electroless nickel immersion gold (ENIG) finish PCB biosensors, such as corrosion and performance variations across multiple PCBs, by using electrodeposited methylene blue (MB) to enhance electroactivity and reproducibility. Moreover, the in-situ synthesis of gold nanoparticles (AuNPs) on acid-functionalized multi-walled carbon nanotubes (MWCNTs) led to achieving a detection limit of 15.79 ng/mL for myeloperoxidase (MPO), an early cardiovascular biomarker. While this capillary-driven microfluidic design is highly cost-effective and well-suited for point-of-care applications, challenges remain in optimizing anti-fouling performance and addressing cross-sensitivity for multi-biomarker assays. This demonstrates the challenges of Lab-on-PCB diagnostic technologies, and its potential for scalable, cost-effective biomarker diagnostics.

**Fig. 10:**
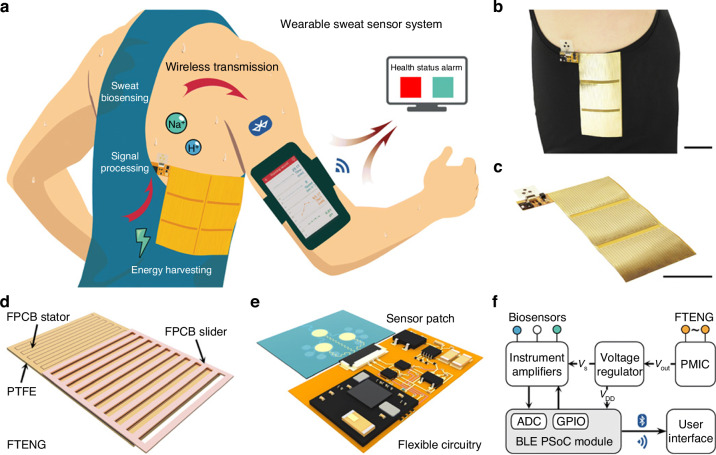
Battery-free freestanding triboelectric nanogenerator (FTENG)-powered wearablesweat sensor system (FWS). **a** Schematic of the FWS^3^, which combines human motion energy harvesting, signal processing, microfluidic sweat biosensing, and Bluetooth wireless data transmission for real-time health tracking. **b**, **c** Optical images of the FPCB-based FWS^3^ worn on the side torso, with a scale bar of 4 cm. **d** Schematic of the FPCB-based FTENG featuring a grating slider and interdigital stator. **e** Schematic of the FTENG-powered wearable sweat sensor system showing the microfluidic sweat sensor patch connected to the flexible circuitry. **f** System-level block diagram illustrating power management, signal transduction, processing, and wireless transmission from the FTENG to the biosensors and user interface. Reproduced from ref. ^183^, copyright 2020, with permission from Science Advances

Glucose sensing, a critical biomarker in diabetes monitoring, has also been extensively explored on Lab-on-PCB platforms, utilizing a range of detection mechanisms including electrochemical^38,105,187–191^ interdigital capacitor (IDC)^192^, using microwave mechanisms^193,194^, and field-effect transistor (FET) approaches^127^. For instance, Song et al. ^183^ developed a battery-free wearable device that combines a Lab-on-PCB-based triboelectric nanogenerator with Bluetooth-enabled sweat sensors. As shown in Fig. 10, this system integrates a microfluidic sweat biosensing platform with flexible circuitry, enabling real-time, non-invasive glucose monitoring. The figure presents the overall schematic of the device, including its energy harvesting mechanism, microfluidic sensor patch, and wireless transmission mechanism, highlighting the potential for non-invasive glucose monitoring. Such platforms are promising for continuous health monitoring.

Advances in Lab-on-PCB devices showcase their adaptability for detecting various pollutants and contaminants in environmental and food monitoring contexts, leveraging multiple sensing techniques such as optical, capacitive, and electrochemical sensors. Optical sensors^60^^,195,196,197^, for instance, are highly suitable for gas-phase analytes such as carbon dioxide, where they are used to monitor production processes in industries like wine and beer^195^, as well as contaminants like airborne nanoparticles^196^, and ultrafine particles^60^. Capacitive sensing has shown potential for quantifying food preservatives like sulfite^198^ and detecting biofilm formation, which is crucial for ensuring food safety^199^. Lab-on-PCB sensors have also demonstrated efficacy in detecting trace metals using electrochemical and SAW sensors, including mercury^114^, and microplastics and algae^132^. These applications highlight the versatility of Lab-on-PCB platforms in both environmental and food safety monitoring applications.

**Fig. 11:**
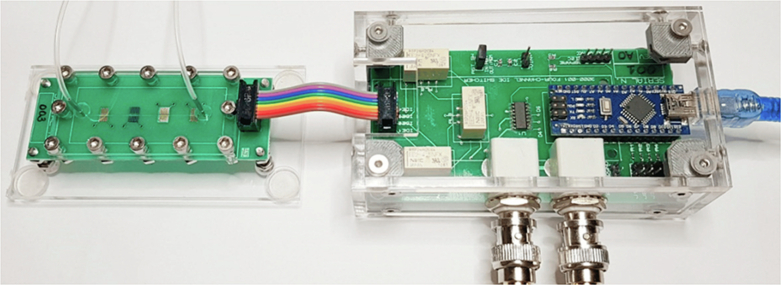
Final device for rapid impedance measurements: impedimetric e-tongue in a single microchannel (left) connected to the digitally controlled analog multiplexing system (right). The device features four collinear, gold-plated interdigitated electrodes (IDEs) embedded on a printed circuit board (PCB), with three electrodes modified with distinct nanostructured materials and one left bare. These sensing units are housed within a single straight microfluidic channel, enabling rapid and automated data acquisition. The multiplexing system, controlled by an Arduino® Nano microcontroller, routes signals from each IDE to a pair of coaxial connectors, minimizing manual errors and signal cross-talk. Reproduced from ref. ^130^, copyright 2020, with permission from MDPI

Another notable application of Lab-on-PCB sensing platforms is in the development of electronic tongues (e-tongues) for distinguishing among flavors and chemical compositions in different samples^130^^,135,200^. For example, Braunger et al.^130^ created an e-tongue based on an array of interdigitated electrodes. As shown in Fig. 11, the device consists of four collinear, gold-plated interdigitated electrodes embedded in a single microfluidic channel, with three electrodes modified using distinct nanostructured materials to enhance selectivity (left). The system, coupled with a digitally controlled multiplexing unit (right), enables automated impedance measurements, reducing manual errors and signal cross-talk. These design elements allow for precise detection of variations in sucralose-based sweeteners, facilitating successful differentiation through principal component analysis. This setup allows for efficient, high-throughput impedance measurements across different analytes, with the system automatically performing three independent measurements per analyte to ensure statistical reliability. This method holds potential for food quality monitoring and safety assurance, offering a rapid, cost-effective way to analyze food compositions and detect other substances, such as adulterants or contaminants in real-time.

In summary, Lab-on-PCB-based sensors exhibit substantial potential in areas such as biosensing, environmental monitoring, and food safety. Their inherent advantages–cost-effectiveness, adaptability, and integration with existing PCB manufacturing processes–position them well for commercialization. However, the path to market-ready devices will require overcoming key challenges, including improving selectivity in complex matrices, ensuring durability under varied conditions, and more importantly, achieving scalable, reproducible fabrication. Addressing these issues will be essential to fully utilize Lab-on-PCB’s potential and expand its applications in real-world scenarios.

### Emerging applications

Lab-on-PCB technology is rapidly finding its way into diverse commercial sectors, driven by the demand for cost-effective, portable, and scalable diagnostic tools. The integration of microfluidic channels, sensors, and electronics onto a single PCB substrate allows these systems to serve to a wide range of emerging applications. Key sectors experiencing transformative advancements through Lab-on-PCB platforms include aerospace and antenna technology.

Recent advances by Lee et al.^201^ have pushed the boundaries of Lab-on-PCB technology, particularly in the area of micro- and nanosatellite propulsion. By leveraging the cost-effectiveness of standard PCB materials and surface mount technology (SMT), the team has created scalable, manufacturable solid propellant micro-thrusters on the Lab-on-PCB platform, offering efficient and reliable ignition systems. Further studies^202^ introduced a membraneless micro-ignitor within the thruster arrays, enhancing control and resilience. Furthermore, in an innovative “Lego-like” modular assembly approach^153^, they developed reconfigurable monopropellant thrusters with multi-layer PCB stacks for horizontal and vertical thrust, addressing prior issues of scalability, heat management, and durability found in MEMS-based thrusters. This configuration ensures consistent, fixed-position thrust generation, improving reliability for spacecraft control. Furthermore, a significant milestone in Lee’s latest work^149^ is the shared-chamber solid-propellant micro-thruster (SCSPM), which integrates a membrane to enable independent activation of each propellant cartridge. The SCSPM allows multi-mode thrust and extended operation times akin to liquid-propellant thrusters but without tanks or valves, operating solely on electric power. This compact, efficient system promises adaptable, sustained propulsion for future space missions.

Beyond propulsion systems, Lab-on-PCB technology is also being explored for other space-related applications. For example, the development of additively printed sensors^203^, such as humidity sensors created using direct-write printing with nScrypt printers, demonstrates the expanding adaptability of Lab-on-PCB technology. These sensors, fabricated with multilayer capacitive inks, offer enhanced flexibility and mechanical durability, making them suitable for use in the harsh environments encountered in space.

Another emerging technology is the integration of antennas onto PCBs^78,84,88^^,204^. These antennas enable wireless communication for applications like wearable health monitoring and environmental sensing within lab-on-chip environments, with recent designs achieving precise frequency and polarization tuning for dynamic testing^84^. Liquid metal (LM) eutectic gallium–indium (EGaIn; 75% gallium, 25% indium) has the characteristics of nontoxicity, good fluidity, and high conductivity (3.4 × 106 S/m), which lead to its broad application prospect in reconfigurable antenna. This reconfiguration method can avoid the disadvantages of reconfiguration using RF switches and achieve a wider frequency tuning range. Furthermore, these liquid antennas can be designed with soft materials, which are promising in flexible and wearable applications^205^.

For instance, Liu et al.^84^ presented an LM-based antenna using EGaIn that enables advanced polarization and frequency control, achieving a 3 dB axial ratio bandwidth from 2.3 to 3 GHz. However, liquid metals continue to suffer from oxidization-related channel stiction issues and likely to require advanced packaging techniques. In addition, liquid metals exhibit an order of lower conductivity as compared to copper and therefore exhibit a major drawback in power handling and higher frequency applications^204^. In contrast, Dey et al.^78^ present an LM-free antenna that uses a movable metallized plate within microfluidic channels for frequency reconfigurability. The LM-free design increases robustness and power-handling capacity but is limited in tuning range.

**Fig. 12:**
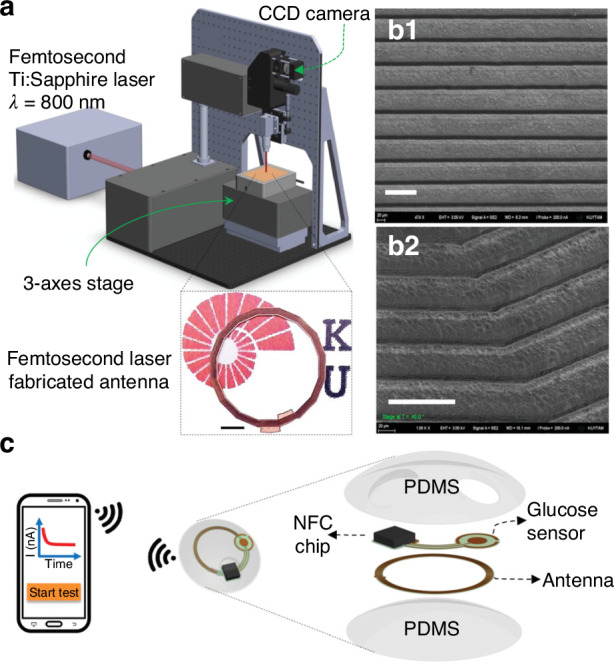
**Femtosecond laser ablation-assisted antenna fabrication**. **a** Diagram of the workstation used to engrave antenna patterns onto a commercially available flexible copper-coated polyimide layer (CCD: charge-coupled device). **b** SEM images of the fabricated antenna (scale bar: 60 µm). **b1** top view showing the uniformity of the antenna lines. **b2** angled view of the antenna highlighting the steep sidewalls of the ablated region, with the sample tilted at 45°. **c** Diagram of the antenna integrated into an SCL for wireless glucose monitoring via a smartphone. Reproduced from^88^, copyright 2022, with permission from John Wiley and Sons

Mirzajani et al.^88^ have also focused on the development of wearable using antennas. They describe the development of a smart contact lens annular ring antenna. Femtosecond ablation technique was used, for the first time, to engrave an NFC antenna structure on a commercially available FPCB layer. The device was integrated with an NFC chip and a glucose sensor to demonstrate its successful operation, as shown in Fig. 12.

These designs collectively highlight the trade-offs in integrating reconfigurable antennas into Lab-on-PCB systems, balancing reconfigurability, durability, and fabrication to support a range of lab-on-chip applications. With emerging applications in fields such as wearable health devices, environmental monitoring, and biomedical sensing, the potential of antenna-integrated Lab-on-PCB systems continues to grow.

## Outlook on standardization

Lab-on-PCB technology has made its first steps into the commercial arena very recently, after demonstrating its scalability and potential for widespread use in laboratory-scale prototypes with increasing confidence, clinical applications and microsystem complexity. Such first ventures include Macias Sensors^206^, biotIP Ltd^207^ and TDX Inc^208^, with a number of PCB electronics manufacturers (i.e. Graphic Plc, Eurotech Plc, Cicor, etc.) capable of offering this type of boards on demand. However, the commercialization of Lab-on-PCB devices is still in its infancy, as this process requires significant investment and time to proceed down the translation pathway next steps of regulatory approvals, certification processes, market adoption and finally patient and society benefit. Academic and entrepreneurial efforts are persistently addressing these hurdles, paving the way for future commercialized products, paving the way for a still largely untapped and pristine market with numerous opportunities and space for innovation in the next years. The ubiquity of PCB manufacturing across the globe allows for this type of innovation to thrive in any part of the Global North or South, removing industrialization barriers often inhibiting ventures in lower and middle income countries.

The previous review of published research proof-of-concept prototypes demonstrates that standardized protocols for the manufacturing of Lab-on-PCB devices is feasible and a desirable next step, encompassing key areas such as material selection, fabrication techniques and parameters, leakage testing, and quality control measures and potentially a technology roadmap similar to the ITRS. Furthermore, the creation of a publicly accessible, open-source repository for microfluidic designs and fabrication methods would facilitate the sharing of knowledge within the community; we have proven the feasibility of this approach with our recent YouTube videos on standardized design^209^. Workshops and committees might be formed to promote the adoption of guidelines and industry standards specifically tailored to microfluidic devices. Different standards could be defined for various microfluidic modalities—such as continuous flow, discrete flow, and digital systems—depending on the specific applications in medical or biotechnological fields.

Zhang et al.^210^ demonstrate the use of Flui3d, an open-source interactive design platform for 3D-printed microfluidic devices. Targeting the challenging and complex task of designing microfluidic networks, Flui3d offers standard parameter component library, multi-layer design support, and the ability to design and configure microfluidic devices without the need for specialized knowledge. Integrating a Design-for-Manufacturing (DFM) function, the software facilitates seamless fabrication of the designed microfluidic devices using commercial consumer-grade printers. Despite the advances in standardized test protocols in microfluidics, none of the approaches to date have addressed the standardization of designing unified microfluidic and electronic structures. The unification of electronics and microfluidics manufacturing within the PCB industry mandates a corresponding unification in the design phase. Thus, the adoption of standard PCB industry CAD software to design the microfluidic structures of the Lab-on-PCB platform has become a recent ambition^3^. Merging the electronics design with the microfluidics design within a single CAD platform will facilitate improved communication with manufacturing facilities and enable the seamless implementation of designs into the production phase.

Another rather recent adoption to target the complexity of designing and controlling microfluidic systems, and hence promote their integration for Lab-on-PCB devices, is the analogy of microflow to an electric circuit. In fluid mechanics, the Hagen-Poiseille explains the hydraulic resistance of pressure-driven flows through circular channels. Analogous to an electronic circuit, this behavior can be explained by Ohm’s Law, which describes the current and voltage drop through a resistive conductor. The pressure drop explained by Hagen-Poiseille can be related to the voltage drop of Ohm’s Law, the volumetric flow rate to current and the hydraulic resistance to electric resistance. These comparisons apply for laminar, viscous and incompressible flows. The analogy, however, can be extended further to include active components, with the capacitance and inductance being analogous to hydraulic compliance and inertia, respectively. Hydraulic capacitance is defined as the change in volumetric flow over the change in pressure, which can be explained by the elasticity of the microchannel.

Designing pressure driven microfluidics via the electric analogy is reviewed rigorously by Oh et al.^211^. Pressure and flow division circuits were designed analogously to voltage and current division, while using Kirchhoff’s law the approximate boundary width of two incoming streams was estimated. Knowing the boundary widths of the two streams, the length of the channel can be designed accordingly to promote mixing at low Reynold numbers. A. Olanrewaju et al.^212^ go a step further, adopting the terminology Capillaric Circuits (CC), reflecting the experimental progress as a conceptual terminology approach. CCs elements discussed include retention valves, vents, reaction chambers, delay valves, etc and a symbolic representation of CCs are proposed.

Vasilakis et al.^144^ designed a cascaded PCB-based serial diluter based on the electric circuit analogy. Passive microfluidics were fabricated on a PCB substrate via the use of dry film photoresist. By correlating the volumetric flow rate to current, pressure drop to voltage and the different microfluidic sections as hydraulic resistances, the microchannel lengths and widths were optimized based on the desired flow rates. They argue that the design of complex microfluidics based on simplified equations for hydraulic equations is accurate only if the microchannel cross section is the same throughout the whole device and highlight the typical 20% deviation of the actual value of hydraulic resistances using this analogy^211^. Despite this limitation, the approach can be used as the initial step of microfluidic designs.

## Conclusion and future research directions

The evolution of PCB-based lab-on-chip (LoC) technology has progressed from basic individual components to highly integrated and self-sufficient platforms supporting the μTAS approach over the past two decades. Lab-on-PCB devices are particularly suited for diagnostic applications, offering accurate analyte quantification at the point of need. Their advantages include well-established industrial infrastructure, advanced microfabrication capabilities, and seamless electronics integration. With a clarified role in μTAS and growing demand for sample-in-result-out integration, especially during challenges like the COVID-19 pandemic, Lab-on-PCB technology is poised for accelerated development. Its alignment with ASSURED criteria underscores its potential for affordable, sensitive, and accessible diagnostic solutions, bridging scientific innovation and commercialization. The standardization of design and fabrication and the reduction of dependence on peripheral equipment and research laboratory infrastructure are the pillars to progress the field, driven by the end user needs. Cooperation between research labs and the PCB industry must grow and the increasing number of published articles highlights the momentum of the field.

Producing sample-in-answer-out, seamlessly integrated multi-component Lab-on-PCB devices entirely within a PCB factory, without requiring additional laboratory processes can prove to be the much anticipated technological enabler for taking LoCs out of the lab and into the public’s hands. This approach ensures scalability, cost efficiency, and consistency, making the technology accessible to a wider market, following the successful paradigm of the electronics industry. By leveraging the mature and standardized infrastructure of PCB manufacturing, complex microfluidic and sensing components can be seamlessly integrated, eliminating the need for time-consuming post-production steps. This streamlined production model accelerates commercialization and fosters the adoption of Lab-on-PCB devices across diverse applications, from healthcare diagnostics to environmental monitoring.

Nonetheless, the establishment of Lab-on-PCB technology as the go-to standard for commercial microsystems opens new research and innovation horizons towards directions and challenges unforeseen until now.

In order to achieve the next stage of maturity as a commercial technology, there need to be Lab-on-PCB devices addressing significant medical or industrial challenges, like the COVID-19 paradigm, with full integration of the devices within existing medical and/or analytical workflows. This in turns places fundamental scientific challenges for researchers, in developing practical microsystems addressing the end-user performance and usability specifications. It also places commercialization and appropriate stakeholder engagement challenges for innovators, relating to developing convincing business cases, capable of attracting the appropriate level of investment in order to achieve regulatory approval and integration into clinical practice.

Considering that for the majority of clinical applications of interest the bioanalytical protocols involve multi-step assays, higher complexity, multicomponent integration of Lab-on-PCB microsystems comes at the forefront. One would imagine the microsystems community (industry and academia) developing the equivalent of the ITRS roadmap, setting the technology integration nodes for the upcoming years and defining the associated material and process challenges for the academic community. Such a streamlining of research effort could accelerate all facets of the technology, in a manner analogous to the semiconductor industry.

Important scientific challenges that can be foreseen at this stage involve certainly material and process aspects affecting the miniaturization of Lab-on-PCB components and features, aiming for higher degrees of integration. More detailed research and understanding of the effect of currently used materials and processed on the miniaturized assay performance is required, such as the impact of copper contamination and electrode physicochemical characteristics of biosensor performance; core material physicochemical characteristics on biomolecule adsorption or reaction inhibitors; physicochemical processes for reducing such performance limitations; material biocompatibility testing and cell toxicity studies. Considering the significance of long-term stability and self-life in commercial applications, a promising research direction is towards the incorporation of inorganic or synthetic substitutes of organic biorecognition molecules.

Finally, the microsystems community cannot be neglectful of its contribution to the climate emergency and its impact on the environment. Lab-on-PCB technology is the only integration approach proposed to date with existing Life Cycle Assessment, via the e-waste processes and standards followed in practice for conventional electronic PCBs. Nonetheless, widescale adoption of Lab-on-PCB technology would exponentially increase e-waste, especially considering that for numerous clinical applications re-usability is not an option. Research and development of new standards for recycling Lab-on-PCB devices would be required, alongside more fundamental research alongside the PCB materials community for biodegradable cores and more sustainable processes, reducing solid and liquid waste production.
